# Role of densin‐180 in mouse ventral hippocampal neurons in 24‐hr retention of contextual fear conditioning

**DOI:** 10.1002/brb3.1891

**Published:** 2020-10-16

**Authors:** Chong‐Hyun Kim, Seoyul Kim, Su‐Hyun Kim, Jongtae Roh, Harin Jin, Bokyung Song

**Affiliations:** ^1^ Center for Neuroscience Brain Science Institute Korea Institute of Science and Technology Seoul Korea; ^2^ Neuroscience Program Division of Bio‐Medical Science and Technology KIST School Korea University of Science and Technology (UST) Seoul Korea

**Keywords:** CaMKIIα, contextual fear conditioning, densin‐180, hippocampus, memory, retrieval

## Abstract

**Introduction:**

Densin‐180 interacts with postsynaptic molecules including calcium/calmodulin‐dependent protein kinase IIα (CaMKIIα) but its function in learning and memory process has been unclear.

**Methods:**

To investigate a role of hippocampal densin‐180 in contextual fear conditioning (CFC) learning and memory processes, knockdown (KD) of densin‐180 in hippocampal subareas was applied.

**Results:**

First, ventral hippocampal (vHC) densin‐180 KD impaired single‐trial CFC (stCFC) memory one day later. stCFC caused freezing behaviors to reach the peak about one hour later in both control and KD mice, but then freezing was disappeared at 2 hr postshock in KD mice. Second, stCFC caused an immediate and transient reduction of vHC densin‐180 in control mice, which was not observed in KD mice. Third, stCFC caused phosphorylated‐T286 (p‐T286) CaMKIIα to change similarly to densin‐180, but p‐T305 CaMKIIα was increased 1 hr later in control mice. In KD mice, these effects were gone. Moreover, both basal levels of p‐T286 and p‐T305 CaMKIIα were reduced without change in total CaMKIIα in KD mice. Fourth, we found double‐trial CFC (dtCFC) memory acquisition and retrieval kinetics were different from those of stCFC in vHC KD mice. In addition, densin‐180 in dorsal hippocampal area appeared to play its unique role during the very early retrieval period of both CFC memories.

**Conclusion:**

This study shows that vHC densin‐180 is necessary for stCFC memory formation and retrieval and suggests that both densin‐180 and p‐T305 CaMKIIα at 1 ~ 2 hr postshock are important for stCFC memory formation. We conclude that roles of hippocampal neuronal densin‐180 in CFC are temporally dynamic and differential depending on the pattern of conditioning stimuli and its location along the dorsoventral axis of hippocampal formation.

## Graphical abstract

Densin‐180, a postsynaptic molecule, in ventral hippocampal neurons is necessary for the contextual fear memory formation during the critical time window at 1~2 hr postlearning. Densin‐180 also affect the phosphorylation status of CaMKII both at basal state and after contextual fear learning. This study shows that densin‐180 is involved specifically and differentially in contextual fear memory formation and/or retrieval process depending on its septo‐temporal location of hippocampus and pattern of learning stimuli.

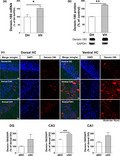

## 1 INTRODUCTION

In mammals, the ability of learning and the storage of memory partly depend on the plasticity of synaptic transmission efficacy between engram neurons and their accompanying excitability changes within the associated neural circuits in the brain (Asok et al., 2019; Bliss & Collingridge, 1993; Langille & Brown, 2018; Lisman et al., 2018; Sossin, 2018). The relative temporal activities between pre‐ and postsynaptic neurons along with surrounding neuromodulators affect the process of synaptic plasticity (Caporale & Dan, 2008; Feldman, 2012). Therefore, as a way of providing cures or preventive treatments of brain disorders with cognitive dysfunctions, it will be important to understand the molecular and cellular mechanisms of synaptic functions in the related regions of the brain (Bourgeron, 2015; Forner et al., 2017; Forsyth & Lewis, 2017; Luscher & Malenka, 2011; Sidorov et al., 2013; van Spronsen & Hoogenraad, 2010; Won et al., 2013).

Densin‐180 (densin) is a neuronal postsynaptic density (PSD)‐enriched protein, existing in both excitatory and inhibitory neurons of mammalian forebrain (Apperson et al., 1996), and is also a member of the LAP [leucine‐rich repeat (LRR) and PDZ (PSD95/discs large/zona occludens‐1)] family, which plays essential roles in sorting of membrane proteins to their target locations and in organizing signaling and structural proteins at cellular junctions including synapses (Bilder & Perrimon, 2000; Ko & Kim, 2007; Santoni et al., 2002). Densin is a cytosolic or membrane‐associated protein, targeting itself to the plasma membrane through its N‐terminal LRR region in transfected cells (Liu et al., 2013; Thalhammer et al., 2009). PSD of excitatory glutamatergic synapse contains many proteins that can initiate diverse signal transduction pathways for the functions of postsynaptic neurons (Sheng & Hoogenraad, 2007). In brain samples, PDZ domain of densin interacts with α‐actinin, Ca_v_1 channels, and MAGUIN 1 (Jenkins et al., 2010; Ohtakara et al., 2002; Sahu et al., 2017; Walikonis et al., 2001; Wang, Stanika, et al., 2017), and C‐terminal domains of densin interact with CaMKIIα, Shank family proteins, and δ‐catenin/NPRAP (Izawa et al., 2002; Jiao et al., 2011; Quitsch et al., 2005; Robison et al., 2005; Strack et al., 2000). Densin plays a role in growth or morphological changes of neuronal processes and spines together with Ca_v_1 channels and shank proteins (Quitsch et al., 2005; Stanika et al., 2016; Vessey & Karra, 2007). Since densin, CaMKIIα, and Ca_v_1 channels interact with each other, densin may be related to the intracellular Ca^2+^ signaling pathway, which is important for synaptic plasticity and morphological changes of neuronal processes (Wang, Marks, et al., 2017). Densin null KO mice have revealed their behavioral abnormalities and impaired learning and memory in object recognition and object location recognition tests (Carlisle et al., 2011). In addition, densin KO hippocampal culture neurons show abnormal synaptic spine morphology and impaired NMDAR‐ and mGluR5‐dependent DHPG‐LTD, while it has normal synaptic transmission, LTP, and CaMKIIα in PSD. Moreover, densin KO reduces the basal level of p‐T286 CaMKIIα but enhances the activity‐dependent phosphorylation of T286 site without changing the synaptic CaMKIIα level. These KO results suggest that densin may be involved in the cognitive brain function.

Among densin‐interacting proteins, CaMKIIα has been extensively studied as a key molecule for synaptic plasticity and memory (Kim et al., 2016; Lisman et al., 2012; Shonesy et al., 2014; Zalcman et al., 2018). p‐T286 CaMKIIα is the autophosphorylated active form which is important for LTP/LTD induction (Buard et al., 2010; Giese et al., 1998; Lengyel et al., 2004), AMPAR function (Barria et al., 1997; Herring & Nicoll, 2016; Opazo et al., 2010), and CFC learning and memory process (Buard et al., 2010; Irvine et al., 2005; von Vertzen & Giese, 2005). Inhibition of dendritic localization of CaMKIIα mRNA, resulting in significant reduction of PSD CaMKIIα, impaired one day CFC memory and late LTP without affecting the short‐term memory and early LTP (Miller et al., 2002). In addition, phosphorylation of T286 site of CaMKIIα enhances the interaction with densin, but phosphorylation of S1397 of densin by CaMKIIα inhibits its binding (Jiao et al., 2011; Walikonis et al., 2001). Furthermore, the autonomous p‐T286 CaMKIIα activity is inhibited by the phosphorylation of T305/306 sites or by densin binding (Colbran & Soderling, 1990; Hanson & Schulman, 1992; Hashimoto et al., 1987; Jiao et al., 2011; Lou & Schulman, 1989; Patton et al., 1990). Therefore, understanding the relationship between densin and CaMKIIα seems crucial for unraveling the synaptic molecular signaling processes of learning and memory mechanism.

In this study, we have focused on a role of hippocampal densin in the contextual fear memory process (Anagnostaras et al., 2001; Gewirtz et al., 2000; Sanders et al., 2003). We have adopted a loss of function strategy with a short‐hairpin RNA (shRNA) of densin in adeno‐associated virus (AAV) to characterize functions of vHC or dorsal hippocampal (dHC) densin during CFC learning and memory formation. The results suggest that the amount of vHC densin about 1 hr after stCFC learning is a crucial factor for the stabilization of CFC memory formation. We further find that stCFC learning causes temporally dynamic and differential modulations of phosphorylation status of both T286 and T305 sites of CaMKIIα, and densin KD reduces the amount of either phosphorylated form of CaMKIIα.

## 2 MATERIALS AND METHODS

### 2.1 Animals

All animal experiments in this study were carried out in accordance with the guidance of the principles in the care and use of experimental animals which was set by the Animal Care and Use Committee of Korea Institute of Science and Technology (ACUCK), and the protocols were approved by ACUCK. All animals are initially produced and kept in specific pathogen‐free facility, and then animals for experiments are transported to and kept in the general mouse room during the experimental period. Only experimenters are allowed to enter the mouse room, and environmental noises are avoided. Each cage contains less than four animals at most. Animals were kept under temperature‐, humidity‐, and light‐controlled conditions (23–25°C, 12‐hr light/12‐hr dark cycle). All experiments were done during light cycle. Mice were given ad libitum to food and water, and the body weight of mouse was measured before surgery, during testing, and at the time of sacrifice. For all experiments, male B6129 F1 mice generated by mating two genetic backgrounds (C57BL/6J, MGI:5657312 and 129/sv, MGI:5658424) were used. All mice were treated in the same way during handling, surgery, and recovery periods, and their body weights were measured regularly. Mouse surgery was done between 8‐ and 9‐week‐olds, and mouse handling for 3 min/day was started at least a week later. 5 days of handling was made before starting behavioral experiments. Mouse condition and its behaviors seemed to recover closely to those of naïve mouse ~10 days after surgery, including the handling period.

### 2.2 Design and construction of densin shRNA vector and virus

To produce AAV for knocking down densin in neurons, shRNAs targeting densin were designed and a set of oligonucleotides was chosen (top, 5ʹ‐TTTCATATTGGATGATAGTAAGAGGTCCTTCCTGTCAGACCTCTTACTATCATCCAATATATTTTT‐3ʹ; bottom, 5ʹ‐CTAGAAAAATATATTGGATGATAGTAAGAGGTCTGACAGGAAGGACCTCTTACTATCATCCATATG‐3ʹ). A vector carrying pAAV‐U6‐promoter and enhanced green fluorescence protein (eGFP, from DiLeone Lab) was used for delivering the densin shRNA, which was subcloned using *SapI* and *XbaI* restriction enzymes, and making the KD virus, AAV_KD‐GFP_. As a control, an AAV‐containing eGFP was made (AAV_Ct‐GFP_).

### 2.3 AAV production and purification

For viral production, HEK293FT cells were cultured in ten plates of 150 × 25‐mm cell culture dishes (SPL #20150) up to 80%~90% confluency. And then cells were transfected with pAAV‐densin shRNA vector, pHelper, and RC plasmids using PEI (Polysciences, Inc. #23966), while pAAV‐GFP was transfected to other ten plates to produce the control AAV virus. After 3 days of transfection, cells were collected, pelleted by centrifugation, re‐suspended in freezing buffer (0.15 M NaCl and 0.05 M Tris, pH 8.0) for 10 min, and then immediately thawed in 42°C for 10 min with shaking. After two cycles of freeze–thaw process for the proper lysis of cells, the mixtures being treated with benzonase (0.5 μl/ml; Sigma, #E1014) were incubated at 37°C for 1 hr and cell debris was removed by centrifuging at 3,580 *g* at 4°C. The supernatants were flown onto HiTrap Heparin columns (GE Healthcare), which were then washed with cold 5‐ml PBS‐MK (1‐mM MgCl_2_, 2.5‐mM KCl in DPBS). Viruses caught in heparin column were eluted by flowing PBS‐MK NaCl (PBS‐MK plus 0.5 M NaCl). The virus solution was concentrated by using Amicon 100 K centrifugal filter (Millipore, # UFC910096) up to 150 μl and stored at −80℃.

### 2.4 Stereotaxic surgery for viral injection

Mouse was anesthetized by injecting 2% avertin (20 μl/g) i.p., and the head was fixed in a stereotaxic apparatus (Stoelting). AAV_KD‐GFP_ or AAV_Ct‐GFP_ (1 × 10^9^ viral genomes) was delivered using an injection syringe (705RN 50 μl SYR, Hamilton Company) at a constant speed of 0.1 μl/min using a microsyringe pump controller (Micro4, World Precision Instruments). The viral injection site of dHC or vHC was targeted with the following coordinates: for vHC, AP −2.8 mm, ML ± 3.0 mm, DV −4.0 mm, and for dHC, AP −2 mm, ML ± 1.5 mm, DV −1.85 mm relative to the bregma. The total injection volume was 2.0 μl for each region. The injection needle was removed 10 min after the end of delivery of viral vector solution. There was no weight difference between control and KD virus‐injected mice groups (Figure S3A).

### 2.5 Contextual fear conditioning (CFC) test

The procedure for testing CFC memory was in accordance with the method of McKinney and Murphy (2006). Fear conditioning experiments were done with a chamber package (Med‐Associates, #MED‐APA‐D1R), whose dimension is 21 × 16 × 20 cm and floor stainless‐steel grid‐bar diameter and spacing are 3 mm and 5 mm, respectively. ENV‐410B was used as stimulation source and ENV‐412 for scrambled shocks. A light bulb and a fan were located inside the chamber, and the intensity of light bulb illumination was 30 ~ 50 lux. After 5 days of handling, mouse home cage was transported and located close to the recording chamber. Each mouse was carried with hands to the chamber for CFC testing. After CFC trial, a home‐made tissue box containing some home cage bedding (Aspen Shavings, kiln‐dried hardwood bedding) was used to carry the mouse back to the home cage. The inside of chamber apparatus including grid bars was cleaned with 70% EtOH, distilled water, and tissue before and after each mouse usage. CFC learning and memory test comprises totally three days of training and testing period. Freezing behavior of mouse is defined when the whole body of mouse including tail does not move more than 1 s and blind observers measure the freezing time off‐line by analyzing video with a custom‐written software. For CFC test, a mouse is placed on the grid bars of the chamber for 3 min for exploration to learn the context (1st training day, Day 0). At the end of 3‐min exploration period, one electrical foot‐shock (0.5 mA/2 s) is delivered to the mouse through the grid bars, and then the mouse is immediately returned to the home cage. On the 2nd day (Day 1), 24 hr after the 1st training, the mouse is placed again in the same chamber for 3 min of exploration and the freezing during the exploration is quantified as stCFC memory. To do double‐trial CFC (dtCFC), the second conditioning trial is made at the end of the test period of Day 1 and then the mouse is returned to the home cage. On the 3rd day (Day 2), 24 hr after the dtCFC training, the mouse is moved to the same chamber and freezing behavior is recorded for 3 min as dtCFC memory test.

### 2.6 Open field test

Open field test is made in a white acrylic box, 40 (H) × 40 (W) × 40 (D) cm, where the top is open. The box chamber is illuminated with indirect white light (12 lux). Mouse is initially placed on the center of box, heading toward wall, and mouse behavior is recorded with video. Mouse explores the chamber freely for 30 min and then is returned to home cage. The chamber is wiped cleanly with 70% EtOH and distilled water during interexperimental intervals. For analysis, bottom surface area of the chamber was invisibly divided into 16 squares and the area of inner four squares was defined as the central area. EthoVision XT 14 (Noldus Information Technology, Inc.) is used to analyze the total moving distance and the time spent in the central area.

### 2.7 Elevated plus maze experiment

Mouse is habituated for 30 min in the experimental room (23 ~ 25°C, 30 lux) before the experiment. The elevated plus maze consists of four acrylic arms in plus (+)‐shape and 50‐cm‐long four metal legs. Two open white arms without walls and another two black arms enclosed with walls (each arm, 5.5 × 30 × 18 cm) are alternate with each other, having the same type of arms direct opposite. Sawdust bedding is spread over arm floors to prevent mouse slippage and to detect mouse in video under black and white floor backgrounds. Arms including walls are cleaned, and new sawdust is used for each mouse trial. Mouse is initially placed on the crossing‐center of the maze for video‐recording its 5‐min exploration and then returned to home cage. The video is monitored off‐line to analyze which arm is chosen, and the latency of first entrance to open arms and the travel distance and time spent in open arms are analyzed with EthoVision XT 14. The anxiety level is calculated as the percentage of the number of mouse whole‐body entrance into the open arms over the total entrance number into either open or closed arms during exploration.

### 2.8 Quantitative real‐time PCR for mRNA quantification

Mice for biochemical analysis, like qRT‐PCR and western blot, were killed by the cervical vertebral dislocation method. After collecting cells in vitro or extracting hippocampal tissues of mice brain, samples were lysed with RNA isolation solution (GeneAll^®^ RiboEx™). Total RNAs from cell lysates were isolated using the GeneAll^®^ Hybrid‐R™ Total RNA kit. RNAs were mixed with SYBR solution (SensiFAST™ SYBR Hi‐ROX Mix 2x, Bioline) and 10 pmol primers for targeting densin (*Lrrc7*) and GAPDH (glyceraldehyde 3‐phosphate dehydrogenase) (densin; forward 5ʹ‐GGACGGTGCTTAATTCAAA CG‐3ʹ, reverse 5ʹ‐CCACCGCTG ATA CTAAATCCA‐3ʹ; GAPDH forward 5ʹ‐ACCCAGAAGACTGTGGATGG‐3ʹ, reverse 5ʹ‐CAC ATTGG GGGTA GGAACAC‐3ʹ, MBiotech, Inc.). Quantitative real‐time PCR (qRT‐PCR) was conducted using a real‐time PCR system (Applied Biosystems) (1 cycle at 50°C for 2 min/95°C for 10 min and 40 cycles at 95°C for 15 s/60°C for 1 min).

### 2.9 Western blot assay

To examine temporal changes of densin expression from vHC or dHC area following stCFC, mice were sacrificed at 1 min, 15 min, 1 hr, and 24 hr after stCFC training. Hippocampus was extracted within 90 s after each time point of behavioral tests in ice‐cold 0.1 M PBS with 1x phosphatase inhibitor cocktail (Xpert Phosphatase, GeneDEPOT, USA) and protease inhibitor cocktail (Xpert Protease, GeneDEPOT, USA). Both vHC and dHC samples were made by cutting the bending domain of each extracted hippocampus and were briefly stored in 1.5‐ml microtube on dry ice before brain homogenization. Homogenization of brain samples was processed on ice with protein extraction buffer (RIPA buffer, 150 mM NaCl, 1% NP‐40, 0.1% SDS, 50 mM Tris‐HCl pH 7.5, 5 mM EDTA, 1 mM PMSF). Homogenized samples in the RIPA buffer were suctioned and pushed back three times with 1‐ml syringe, and then ultra‐sonication of samples was also repeated three times (5‐s sonication and 15‐s waiting time). The homogenate was centrifuged at 13,475 *g* for 20 min at 4°C. The supernatant was used for western blotting. Protein concentration of each sample was determined by Tecan multi‐functional microplate reader (Infinite M200 Pro, Switzerland), and then the concentration was adjusted by dilution with protein extraction buffer and 5× SDS solution (GeneAll Biotechnology Co, LTD, South Korea). The final sample (20 μg/lane) was boiled for 5 min at 98°C and then loaded to SDS‐polyacrylamide gel electrophoresis (8% of acrylamide/0.27% *N, N*ʹ‐methylene‐bis‐acrylamide resolving gel) for 20 min at 80 volt in the stacking gel and for 1 hr 10 min at 120 volt in the resolving gel. Proteins were transferred to polyvinylidene fluoride transfer membranes (GE Healthcare) for 80 min at 280 mA on ice. The blots were washed three times for 10 min with TBS‐T (48 mM Tris, 39 mM glycine, 0.0375% SDS, 20% methanol in TBS‐T; plus 0.05% Tween‐20 in TBS, pH 7.4) and blocked with 5% bovine serum albumin (BSA) in TBS‐T for 1 hr at room temperature (RT). For the administration of primary antibodies, blots were incubated in TBS‐T with primary antibodies (polyclonal anti‐rabbit densin, 1:1000, Santa Cruz, Inc., AB_2137760 & 2868505; monoclonal anti‐mouse p‐T286 CaMKIIα, 1:1000, Thermo Fisher Scientific, USA, Ab_325402; polyclonal anti‐rabbit p‐T305 CaMKIIα, 1:1000, Fitzgerald, USA, AB_2868506; monoclonal anti‐mouse CaMKIIα, 1:2000, Millipore, AB_309787; polyclonal anti‐rabbit‐GAPDH, 1:1000; Santa Cruz, Inc., AB_10167668) in TBS‐T plus 1% BSA at 4°C for overnight while shaking at 20 rpm. After three times of 10‐min wash in TBS‐T buffer, the blots were incubated in the secondary‐conjugated‐horseradish peroxidase (HRP) (goat anti‐rabbit IgG, goat anti‐mouse IgG; 1:1500; Santa Cruz, AB_631736, AB_631746, AB_631747) in TBS‐T plus 1% BSA for 1 hr at RT on shaker and then washed again three times for 10 min each. Protein bands were detected by using enhanced chemiluminescence (Bio‐Image Analysis System ImageQuant LAS4000, GE Life Science). Intensities of all protein bands were analyzed using ImageJ software (NIH, USA, SCR_003070). Negative control bands were tested by probing only with the secondary antibody, which resulted in no detection of bands. The positive protein bands were calculated only when the background intensity of each blot was eliminated. Then, the intensity of each band was normalized over the average value of control vHC samples of the home cage group.

### 2.10 Preparation of brain slice sections

Mice were anesthetized using 2% avertin (20 μl/g, i.p), perfused with 0.1 M phosphatase‐buffered saline solution (PBS, in mM; 137 NaCl, 2.7 KCl, 4.3 Na_2_HPO_4_, 1.47 KH_2_PO_4_; pH 7.4), fixed with 4% paraformaldehyde, and then brains were removed. All brains were postfixed in 4% paraformaldehyde at 4°C for 4 hr and then dehydrated in 30% sucrose in 0.1 M PBS at RT overnight. Brain slices were made as coronal 40‐μm thickness by cryostat (Microm, HM525), and all sections from indicated hippocampal regions were collected in 0.1 M PBS (dHC, from bregma −0.94 mm to −3.40 mm; vHC, from bregma −2.185 mm to −3.64 mm). Several tissues were used for free‐floating immunohistochemistry, and the remaining tissues were stored in 0.1 M PBS/glycine (1:1) solution at −20°C for additional experiments.

### 2.11 Free‐floating immunohistochemistry

Brain slices were washed three times with 0.1 M PBS, permeated with 0.4% Triton X‐100 in 0.1 M PBS for 20 min at RT, and then blocked with 5% normal goat serum and 0.25% Triton X‐100 in 0.1 M PBS for 1 hr at RT. Tissues were incubated with the primary antibody (densin; polyclonal goat anti‐rabbit, Santa Cruz, Inc., 1:200, DAPI; 1:1000, AB_2137763) in 2% normal goat serum, 0.15% Triton X‐100 in 0.1 M PBS for 24 hr at 4°C. Next, tissues were washed three times with 0.1 M PBS, incubated with the Alexa Fluor‐Cy5‐conjugated secondary antibody (goat anti‐rabbit Cy5, 1:200, AB_2868518) for 2 hr at RT. Tissues were then washed three times with 0.1 M PBS, stained with DAPI (1:1000) to stain cell nuclei, for 20 min at RT, and mounted on slides with Glycergel (mounting medium, Dako). Fluorescent images were acquired with a confocal microscope (Olympus). Image samples were made under the same IHC conditions on the same day, and images of all samples made from each Ab or dye were acquired under the same laser lighting conditions. The intensities of blue (405 nm) and red (594 nm) signals were quantified with ImageJ program (NIH, USA).

### 2.12 Statistical analysis

All measurements were presented as means ± *SEM* with two‐tailed Student's *t* test (α = 0.05) unless otherwise mentioned. Unpaired independent *t* test was used to compare the experimental group with the control group. P_cont_ (t1, t2) indicates the *p* value of *t* test between control data groups at times, t1 and t2. Repeated measure (RM) two‐way ANOVA tests and Sidak's multiple comparison post hoc analysis were used whenever necessary (GraphPad Prism v8.2.1, USA, SCR_002798).

## 3 RESULTS

### 3.1 Higher basal densin expression in ventral hippocampus than in dorsal hippocampus

**FIGURE 1:**
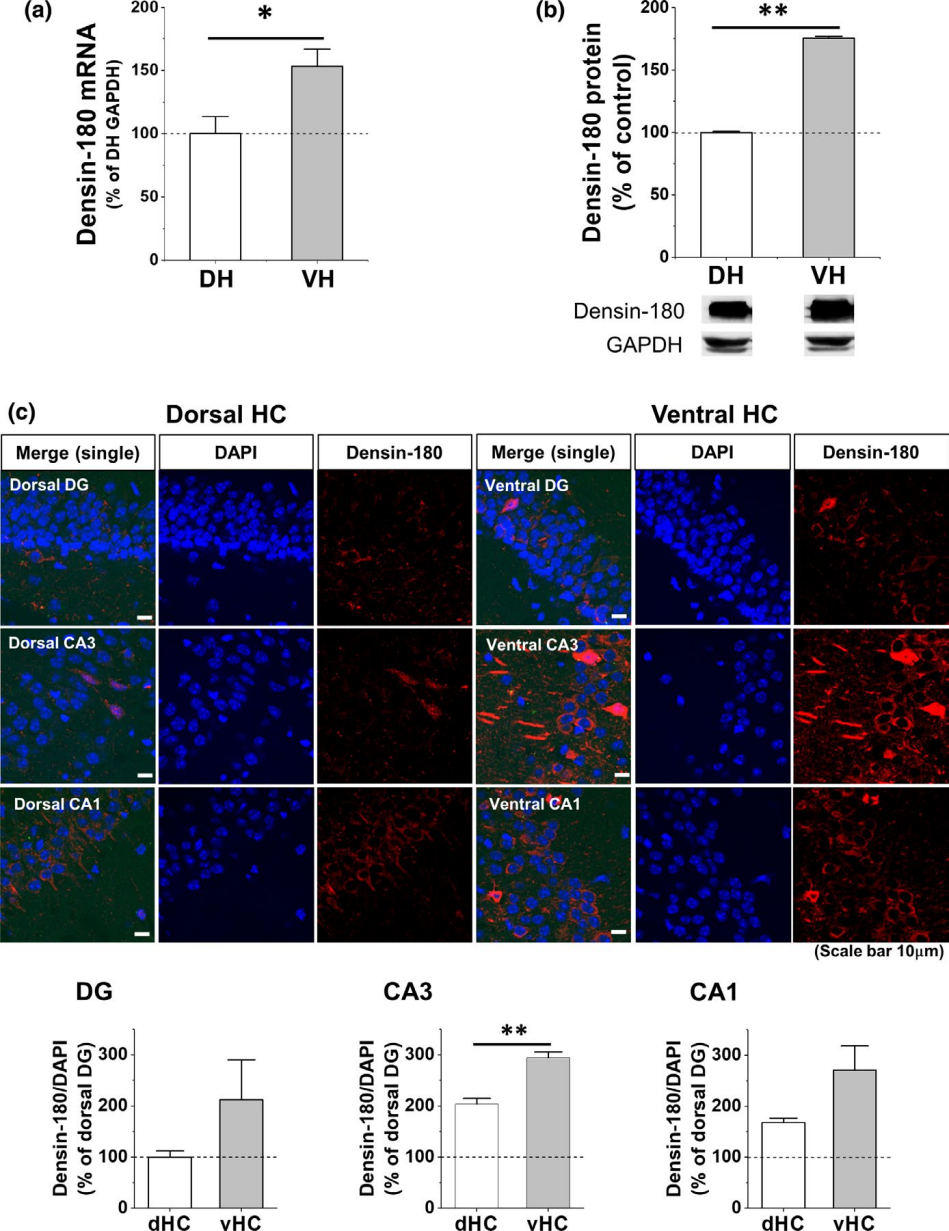
Densin expressions in dorsal and ventral hippocampus of adult mouse brain. (a) Densin mRNA levels in vHC and dHC of WT control mouse brain. d = dorsal, v = ventral (*p < .05; *n* = 12). Two‐tailed *t* test at α = 0.05 unless otherwise mentioned. (b) Densin protein levels in vHC and dHC of WT control mouse brain (**p < .01; *n* = 4). (c) Densin expression in the subdivisions of dHC and vHC. *Top, left*: representative images of each subdivision of dHC. *Top, right*: representative images of each subdivision of vHC. *Bottom*, quantitative plots of fluorescence intensity of densin (red) and DAPI (blue) in each subdivisions of HC. Region of interest is the whole square image (scale bar, 10 μm). 4 tissues/mouse, *n* = 2~3; **p < .01; DG, *p* = .29; CA3, *p* = .0048; CA1, *p* = .59

vHC and dHC are known to be involved in the expression, consolidation, or retrieval of CFC memory (Bast et al., 2001; Kheirbek et al., 2013; Kim & Cho, 2017; Lee & Kesner, 2004; Quinn et al., 2005; Rudy & Matus‐Amat, 2005; Sutherland et al., 2008; Zhu et al., 2014), and differential expressions of molecules including synaptic membrane proteins along septo‐temporal hippocampal subdivisions are observed either at basal state or after CFC learning (Fanselow & Dong, 2010; Rao‐Ruiz et al., 2015). Therefore, we quantified densin from vHC or dHC of naïve mice separately (Figure 1). We found that the basal mRNA and protein levels of vHC densin were ~53% and ~76% higher than those of dHC densin, respectively (Figure 1a,b). Immunohistochemistry results also showed higher fluorescent intensity of densin in vHC region compared to dHC region (Figure 1c). The results from each subarea of hippocampus of naïve mouse showed that each vHC subarea seemed to express more densin in general compared to its corresponding subarea of dHC, where basal expression of vHC CA3 densin was significantly bigger (~44%) compared to dHC one (Figure 1c bottom). This result suggested a possibility that vHC densin might have a unique role in hippocampal‐dependent learning and memory processes, even compared to dHC densin.

### 3.2 CFC induces a reduction of densin expression in ventral hippocampus selectively

**FIGURE 2:**
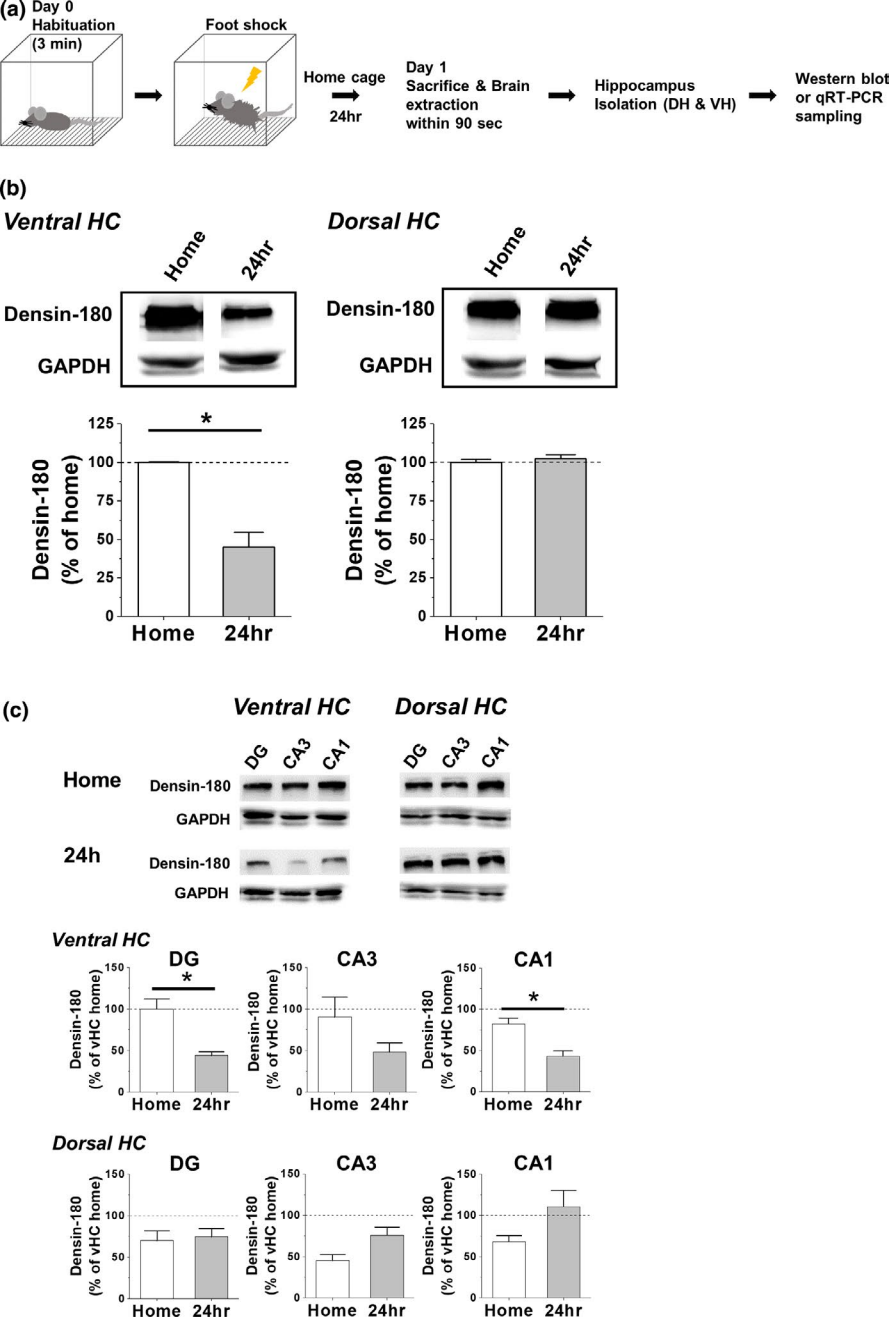
Densin expression in dorsal and ventral hippocampus of naive mouse 24 hr after stCFC. (a) Scheme of tissue sampling process for biological analysis 24 hr after stCFC learning. See Section 2. Two‐tailed *t* test at α = 0.05 unless otherwise mentioned. (b) *Left*, densin expression in vHC of naïve mouse at both basal state and 24 hr after stCFC training (*p = .011; *n* = 4/group). *Right*, densin expression in dHC of naïve mouse at both basal state and 24 hr after CFC training (*p* = .63). (c) Densin expression in each subdivisions of ventral and dorsal HC at 24 hr after stCFC. *Top*, representative western blot images under conditions at both control home cage and 24 hr after CFC learning. *Middle*, quantitative densin intensity measurements in subregions of vHC (vDG, **p = .004; vCA3, *p* = .14; vCA1, *p = .01; *n* = 3~4/group). *Bottom*, quantitative densin intensity measurements in subregions of dHC (dDG, *p* = .54; dCA3, *p* = .065; dCA1, *p* = .14; *n* = 3~4/group)

To find out how CFC stimulus affects the densin expression in hippocampal subdivisions, densin was measured one day after stCFC learning trial (Figure 2a). Since shock or context‐only nonassociative control or viral injection procedure did not affect the basal levels of vHC densin, CaMKIIα, and p‐T286 CaMKIIα, only home cage control was used (Figure S1). vHC densin was reduced by ~55% over that of basal home cage state, while dHC densin level was not changed (Figure 2b). Each subregions of vHC showed overall reductions of densin (~47%–~56%) compared to their home cage controls, where reductions of vHC DG and CA1 regions were significant, while those of dHC subregions were not affected (Figure 2c). The results showed that stCFC learning stimulus downregulated the expression of vHC densin a day later.

### 3.3 VHC densin KD results in the impairment of the contextual fear memory one day later

To study a role of densin in hippocampal neuronal functions, we subcloned a densin shRNA into an AAV vector (AAV_KD‐GFP_) to suppress densin expression and checked the effect of densin KD on the contextual fear memory formation. The results of KD efficiency experiments showed that vHC densin KD reduced both mRNA and protein of vHC densin by ~70% and ~31%, respectively, compared to control samples after 2‐week infection and vHC KD process did not affect dHC densin mRNA and protein levels (Figure S2).

**FIGURE 3:**
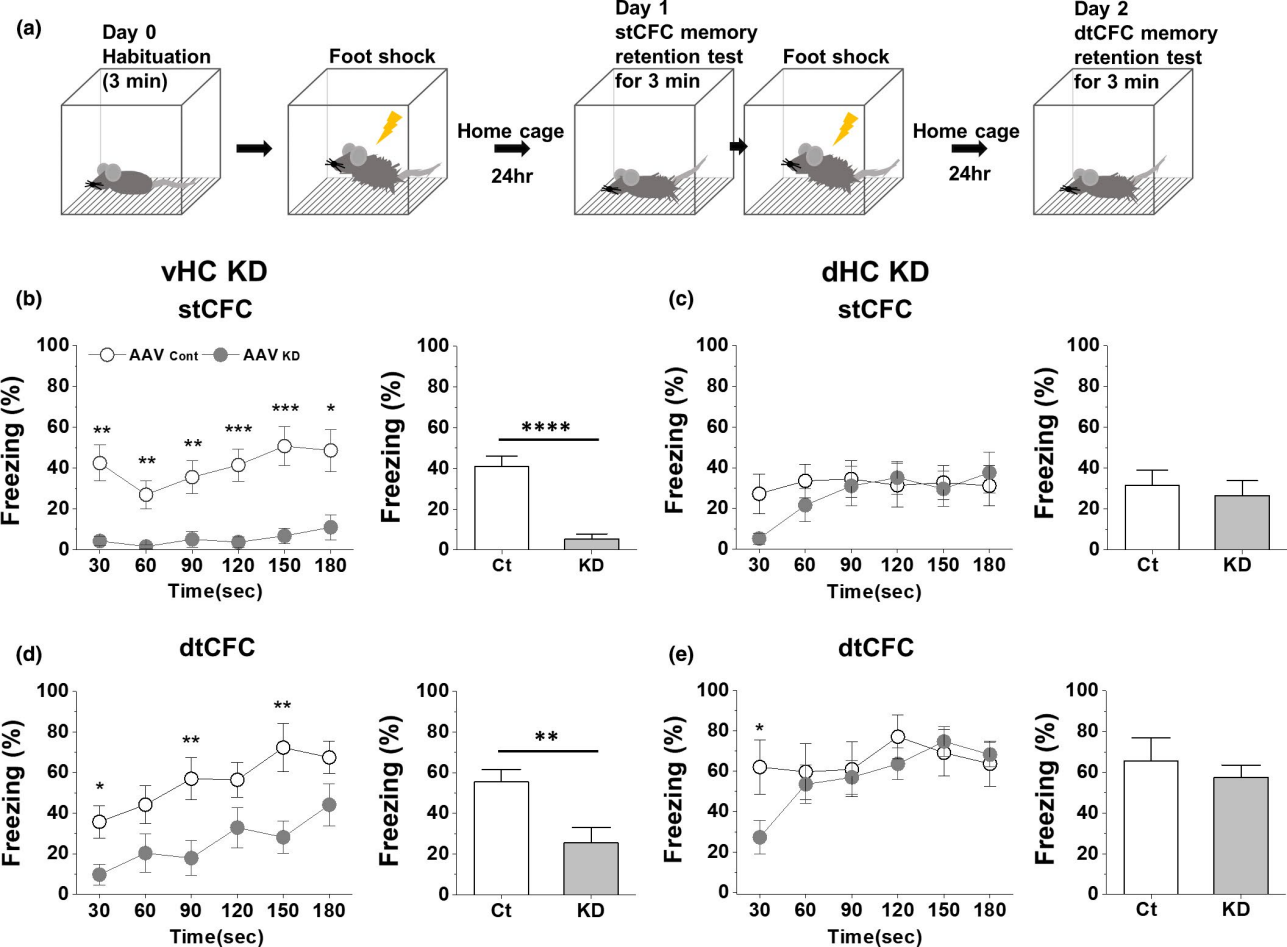
Contextual fear memory retention tests 24 hr after stCFC and dtCFC learning in vHC and dHC densin KD mice. (a) Scheme of stCFC and dtCFC learning and memory retention tests. (b) *Left*, the time course of freezing responses for the 3‐min test period as percentage of total freezing in time one day after stCFC learning in vHC KD mice. Ct = control, KD = densin KD. Two‐tailed *t* test at α = 0.05 unless otherwise mentioned (AAV_Ct_ vs. AAV_KD_, *p_(30 s)_ = .0014, **p_(60 s)_ = .0042, **p_(90 s)_ = .0039, ***p_(120 s)_ = .0006, ***p_(150 s)_ = .0009, **p_(180 s)_ = .0051; *p*_Ct (30 s, 180 s)_ = .65, *p*_KD (30 s, 180 s)_ = .32). RM two‐way ANOVA shows a significant effect of KD (time × densin, *df* = 5, *F* = 0.86, *p* = .5098; time, *df* = 5, *F* = 2.2, *p* = .0918; densin, *df* = 1, *F* = 46, *p* < .0001; group, *df* = 22, *F* = 3.2, *p* < .0001), and Sidak's test shows all significance at all time points (*p* < .05). *Right*, the total average freezing response of vHC densin KD mice one day after stCFC learning compared to GFP‐AAV_Cont_ injection mouse (****p = .00002; *n* = 11/group). (c) *Left*, the time course of freezing responses for the 3‐min test period as percentage of total freezing in time one day after stCFC learning in dHC KD mice (AAV_Ct_ vs. AAV_KD_, *p*_(30 s)_ = .035, one‐tail *t* test; *p*_Ct (30 s, 180 s)_ = .79, **p_KD (30 s, 180 s)_ = .0098). RM two‐way ANOVA shows a significant effect of time (*F* = 3.0, *p* = .024), and Sidak's test shows none significance at all time points. *Right*, the total average freezing response of dHC densin KD mice one day after stCFC learning compared to GFP‐AAV_Ct_ injection mouse (AAV_Ct_, *n* = 9; AAV_KD_, *n* = 11, *p* = .64). (d) *Left*, the time course of freezing responses for the 3‐min test period as percentage of total freezing in time one day after dtCFC learning in vHC KD mice (AAV_Ct_ vs. AAV_KD_, *p_(30 s)_ = .012, **p_(90 s)_ = .009, **p_(150 s)_ = .006; **p_Ct (30 s, 180 s)_ = .0011, **p_KD (30 s, 180 s)_ = .0094). RM two‐way ANOVA shows significant effects of KD (time × densin, *df* = 5, *F* = 1.1, *p* = .3826; time, *df* = 5, *F* = 8.9, *p* < .0001; densin, *df* = 1, *F* = 11, *p* = .0032; group, *df* = 22, *F* = 6.4, *p* < .0001), and Sidak's test shows significance at *t* = 30, 90, 150 s (*p* < .05). *Right*, the total average freezing response of vHC densin KD mice one day after dtCFC learning compared to GFP‐AAV_Ct_ injection mouse (AAV_Ct_, *n* = 7; vHC AAV_KD_, *n* = 7; **p = .006; total percentage of freezing in time). (e) *Left*, the time course of freezing responses for the 3‐min test period as percentage of total freezing in time one day after dtCFC learning in dHC KD mice (AAV_Ct_ vs. AAV_KD_, *p_(30 s)_ = .033; *p*_Ct (30 s, 180 s)_ = .93, ***p_KD (30 s, 180 s)_ = .00077). RM two‐way ANOVA shows significant effects of time (*F* = 7.7, *p* < .0001) and interaction (*F* = 4.4, *p* = .0012), and Sidak's test shows none significance at all time points. *Right*, the total average freezing response of dHC densin KD mice one day after dtCFC learning compared to GFP‐AAV_Ct_ injection mouse (AAV_Ct_, *n* = 6; dHC AAV_KD_, *n* = 8, *p* = .83)

The freezing at basal state was zero and was not affected in either vHC or dHC KD mice (see Day 0 values in Figure S3B–C). Freezing, a behavioral marker for CFC memory formation and its successful retrieval, was measured one day later (Day 1) (Figure 3a). Freezing behavior of control mice from vHC or dHC KD group on Day 1 appeared immediately and was maintained quite stable for 3‐min memory retention test period (open circles, Figure 3b,c, p30*_:180_* = .65 and .79, respectively), suggesting successful acquisition of CFC learning, its subsequent memory formation, and the immediate full retrieval of the memory. In vHC densin KD mice, the average freezing was reduced by ~87% on Day 1 compared to control and there was little progression of freezing behaviors during the test (*p*_KD (30 s, 180 s)_ = .32) (Figure 3b), suggesting failure of either memory formation or memory retrieval. In dHC densin KD mice, the freezing behavior was developed sharply from near zero to the stable level during the test period on Day 1 (*p*_KD (30 s, 180 s)_ = .0098), which did not affect the average freezing level significantly (Figure 3c). The full recovery of freezing behavior within the test period suggests that both the acquisition of stCFC learning and the memory formation are normal in dHC KD mice, but it takes about a minute for its full retrieval, a delay compared to control mice.

### 3.4 Double‐trial CFC reveals different effects of VHC densin KD on CFC memory formation

It has been shown that the inhibitory effects of Ca_v_1.3 deletion or CaMIIα T286A mutation on stCFC memory formation are gone after additional learning trainings like dtCFC (Irvine et al., 2005; McKinney & Murphy, 2006). These suggest that dtCFC may activate extra cellular and molecular signaling pathways or neuronal circuits, which are distinct from those activated by stCFC. Therefore, we checked the effect of densin KD on dtCFC memory formation, shown in Figure 3a. A notable result was that an incremental progress of freezing was observed during the test on Day 2 in either vHC control or KD mice. The kinetic change of freezing in KD mice does not differ from that of control mice (linear regression for the slope, *p* = .646), indicating no effect of densin on the kinetic of the progressive retrieval process (Figure 3d left). However, in terms of the magnitude of freezing, the average freezing in KD mice was smaller by ~54%, suggesting that densin KD does seem to inhibit dtCFC memory formation (Figure 3d right). In control mice, the freezing after stCFC was maintained stable during the test (*p_30:180_* = .65) but the freezing after dtCFC was increased from ~36% to ~67% during the test (*p_30:180_* = .0011) (Figure 3b,d). In KD mice, stCFC freezing was little and stable (*p_30:180_* = .32) and dtCFC freezing was increased from ~10% to ~44% during the test (*p_30:180_* = .0094). As a result, freezing levels at the end of stCFC and dtCFC memory tests in KD mice were smaller by ~78% and ~34% over their controls, respectively. The results suggest that vHC densin plays a crucial role for both formation and retrieval of stCFC memory, while it seems necessary only for the consolidation of dtCFC memory.

### 3.5 dHC densin seems to affect the initial retrieval process of CFC memory

dHC densin KD had little effect on the average freezing level during stCFC or dtCFC memory retention test (Figure 3c,e right). However, there existed a difference in the kinetics of early freezing responses during both memory tests between control and KD mice (Figure 3c,e left). While control mice exhibited stable freezing behaviors during the test, KD mice took a min or so to reach a plateau starting from the smaller initial freezing levels in both tests. In addition, the average freezing level of dtCFC memory test was about twice of that of stCFC memory test in either between control groups or between KD groups, respectively (~66% vs. ~32% in control; ~55% vs. ~26% in KD, Figure 3c,e right). The results suggest that dHC densin is involved in the very early retrieval processes of both stCFC and dtCFC memories, but not in the acquisition or storage of both memories. The similar recovery time course of early freezing behaviors in both tests further suggests that dHC densin plays the same kind of a transient role during the early retrieval process of CFC memories irrespective of the number of conditioning trials.

### 3.6 VHC densin KD mice exhibit the impaired development of CFC freezing behavior 2 hr later

**FIGURE 4:**
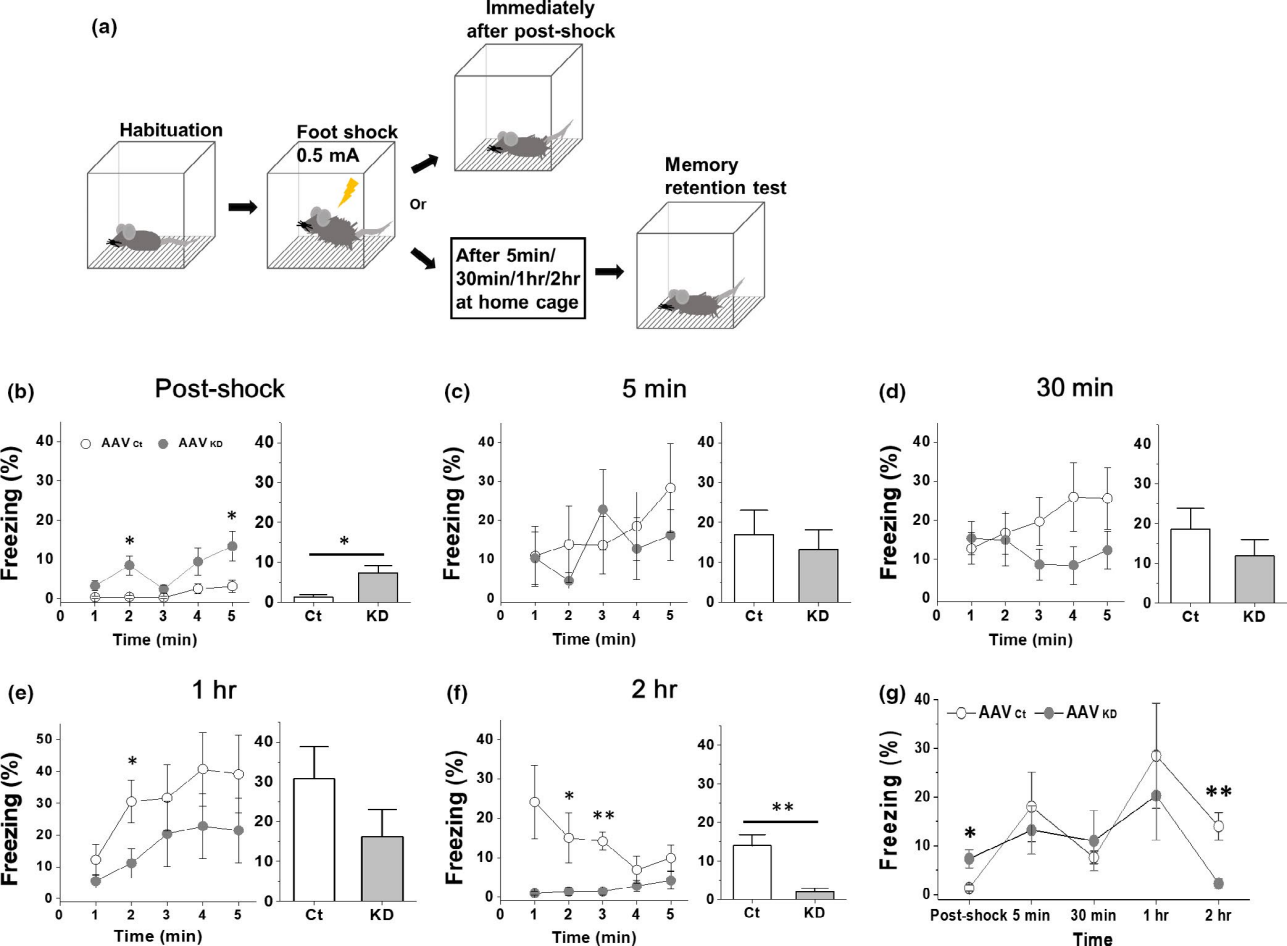
Time courses of acquisition and maintenance of stCFC learning in the vHC densin KD mice. (a) Scheme of freezing tests during stCFC learning and memory acquisition periods. Tests (re‐exposure to the context) were done on the 11th day after densin shRNA AAV injection. Habituation was done for 3 min/day. The acquisition of stCFC learning was measured at the following intervals: zero (immediately postshock), 3 min, 5 min, 30 min, 1 hr, and 2 hr postshock. The test period was recorded for 5 min. Two‐tailed *t* test at α = 0.05 unless otherwise mentioned. (b–f) Freezing responses for 5‐min recording periods at indicated intervals after stCFC learning. (b) Freezing levels measured immediately after stCFC (postshock). Left: AAV_Ct_, *n* = 7; AAV_KD_, *n* = 8, *p_(2 min)_ = .014, *p_(5 min)_ = .036. RM two‐way ANOVA shows significant effects of KD (*F* = 8.54, *p* = .012) and time (*F* = 5.5, *p* = .0067), and Sidak's test shows none significance at all time points. Right: *p = .013451. (c) Freezing levels at 5 min interval. Left: AAV_Ct_, *n* = 7; AAV_KD_, *n* = 8. RM two‐way ANOVA shows no significant effects of KD, time, and interaction, and Sidak's test shows no significant effects at all time points. Right: *p* = .6435776. (d) Freezing levels at 30‐min interval. Left: AAV_Ct_, *n* = 7; AAV_KD_, *n* = 6. RM two‐way ANOVA shows a significant effect of interaction between KD and time (*F* = 4.6, *p* = .0035), and Sidak's test shows no significant effects at all time points. Right: *p* = .35. (e) Freezing levels at 1‐hr interval. Left: AAV_Ct_, *n* = 7; AAV_KD_, *n* = 7, *p_(2 min)_ = .036. RM two‐way ANOVA shows a significant effect of time (*F* = 5.6, *p* = .0048), and Sidak's test shows no significant effects at all time points. Right: *p* = .19). (f) Freezing levels at 2‐hr interval. Left: AAV_Ct_, *n* = 7; AAV_KD_, *n* = 7, *p_(2 min)_ = .022, **p_(3 min)_ = .0037. RM two‐way ANOVA shows a significant effect of KD (*F* = 17, *p* = .0016), and Sidak's test shows a significant effect at 3 min (*p* = .018). Right: **p = .0041. (g) Time courses of development of freezing responses for the 5‐min recording period after stCFC learning (AAV_Ct_ vs. AAV_KD_, postshock, *p_(0)_ = .013, **p_(2 hr)_ < .05). RM two‐way ANOVA shows a significant effect of time (*F* = 5.0, *p* = .0018), and Sidak's multiple comparison shows no significant effects at all time points

Since Day 1 stCFC memory was impaired in vHC densin KD mice, we decided to check whether KD affected the acquisition of stCFC memory. To see the development of memory acquisition, we observed freezing responses at each of the following time points after stCFC: immediately postshock, 5 min, 30 min, 1 hr, and 2 hr (Figure 4a), at which independent groups of mice were used to sample brain tissues for biochemical analysis. The behavioral results are shown in Figure 4b–f where left plot shows the time course of freezing behaviors during each test and the right bar graph shows its average freezing response. The summary plot is shown in Figure 4g. The result showed that vHC densin KD mice failed to retrieve the fear memory around 2 hr postshock, suggesting that vHC densin at 1 postshock is crucial for the stabilization of stCFC memory. Additionally, the immediate freezing response after shock appeared higher in vHC KD mice, for which an explanation was discussed (Figure 4b).

### 3.7 VHC densin KD mice show no significant effects on locomotor activities and anxiety‐like behaviors

**FIGURE 5:**
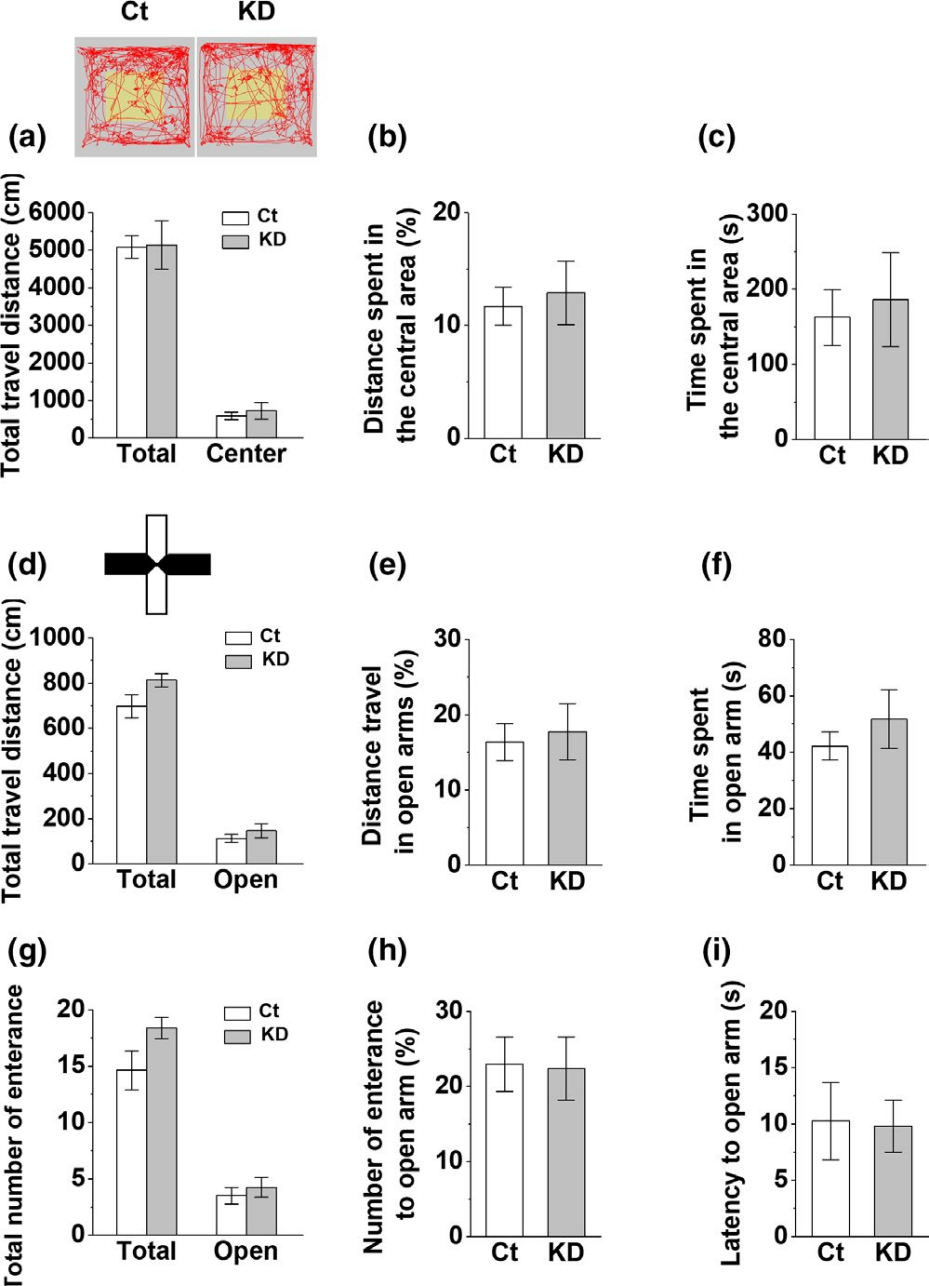
Open field test and elevated plus maze test in vHC KD mice. (a–c) Open field test. *Inset*, example traces of moving activities of control mouse (left) and KD mouse (right). Red trace, moving location; gray square, total open field area; yellow square, central area. (a) Total travel distance inside the open field box (dotted bar, *p* = .94) or within central area (Ct, open; KD, gray; *p* = .59) for 5‐min exploration, *n* = 8. (b) The percentage of travel distance in the central area of the open box (*p* = .72). (c) Time spent in the central area of the open box (*p* = .75). (d–i) Elevated plus maze test. *Inset* is a top view image of elevated plus maze. (d) Total travel distance on the all arms (dotted bar, *p* = .084) and on the open arms only (Ct, open; KD, gray; *p* = .37) for 5‐min exploration. (e) The percentage of travel distance in the open arms (*p* = .37). (f) Time spent in the open arms (*p* = .43). (g) Total number of entrances (dotted bar, *p* = .077) or in the open arms only (Ct, open; KD, gray; *p* = .52). (h) Latency to first entrance to open arms (*p* = .91). (i) Number of entrance to open arms (*p* = .52)

Since vHC is related to anxiety‐like behaviors which may affect the freezing response of CFC memory (Bannerman et al., 2004; Kheirbek et al., 2013; Weeden et al., 2015), it is possible that vHC densin KD in both hemispheres may affect anxiety‐like behaviors. To check whether vHC densin KD changed anxiety levels of mice, we did the open field test and analyzed the total moving distance of locomotor activity and both the travel distance and the time spent in the central area of open field as indicators of anxiety level. We found no significant differences in those three parameters between control and densin KD mice (Figure 5a–c). The elevated plus maze test, another anxiety test, also showed no significant difference in the total travel distance in all arms or in open arms only, the percentage of travel distance and the time spent in open arms, the number of total entrance to all arms, the latency to the first entrance to open arms, and the percentage of entrances into open arms, between control and densin KD mice (Figure 5d–i). Therefore, it seems that vHC densin KD itself does not affect the general locomotor activity and anxiety‐like behaviors of mice.

### 3.8 stCFC causes a biphasic change of VHC densin level in a day

**FIGURE 6:**
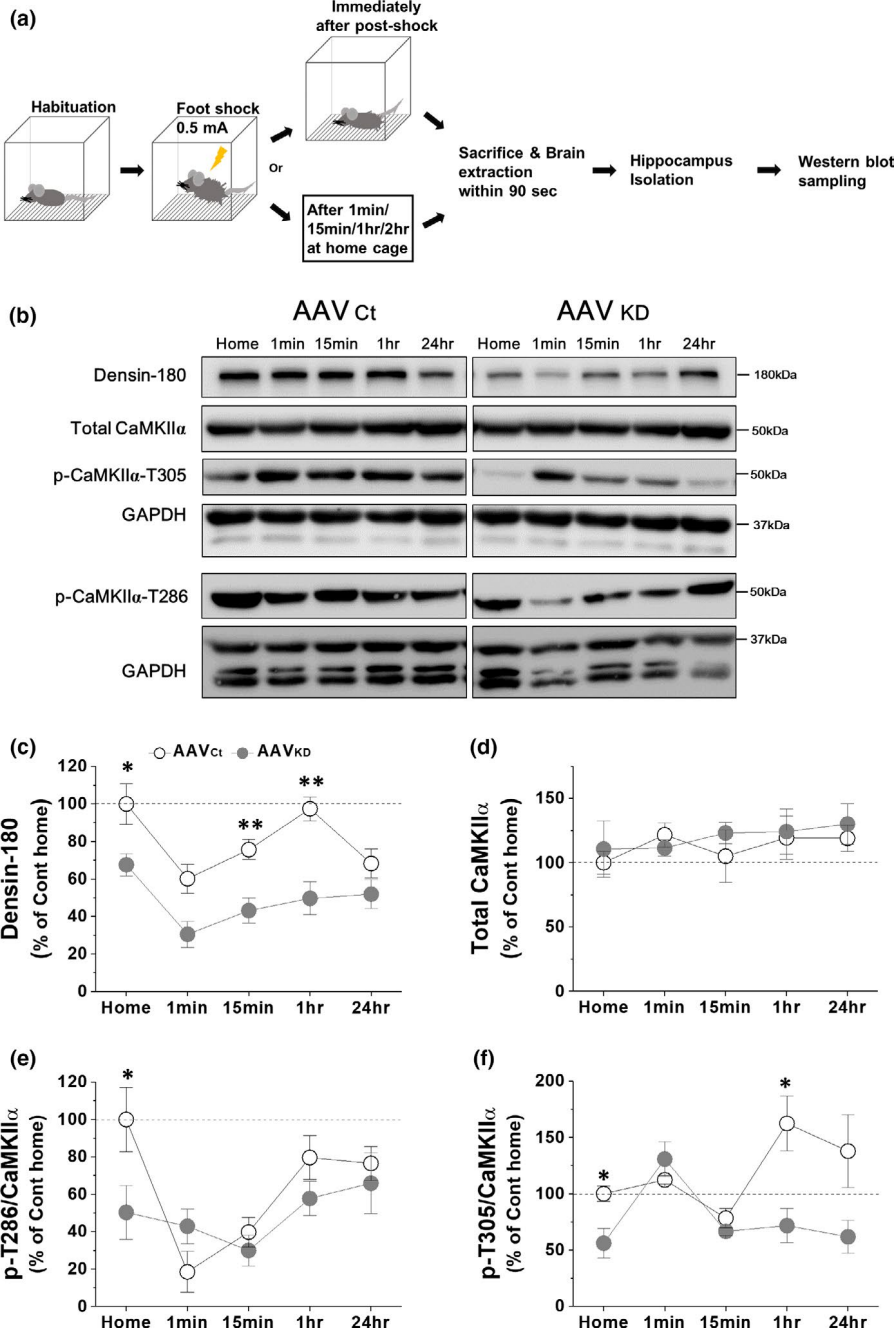
Densin, p‐T286 CaMKIIα, and p‐T305 CaMKIIα after stCFC learning in vHC densin KD mice. (a) Scheme of tissue sampling process during stCFC learning and memory acquisition period. (b) Representative western blot images of densin, p‐CaMKIIα‐T305, p‐CaMKIIα‐T286, total CaMKIIα, and GAPDH from vHC samples of densin KD mice and control GFP‐AAV injection mice at different time points after stCFC learning. The same GAPDH blot was reused for densin, total CaMKIIα, and p‐CaMKIIα‐T305 quantification. *n* indicates mouse number. (c) Time course of changes of densin after stCFC learning in control and KD mice. Densin expression was normalized over the basal expression level of control mice at home cage. Two‐tailed *t* test at α = 0.05 unless otherwise mentioned. *p < .05; **p < .001 (densin; AAV_Ct_ vs. AAV_KD_, *p_(home; Ct, vKD)_ = .023, **p_(15 min; Ct, vKD)_ = .0080,**p_(1 hr; Ct, vKD)_ = .0041) [*p* values in AAV_Ct_ groups, *p_(home, 1min)_ = .02362, *p_(home, 24 hr)_ = .040, **p_(1 min, 1 hr)_ = .0098, *p_(15 min, 1 hr)_ = .039, *p_(1 hr, 24 hr)_ = .029; *p* values in AAV_KD_ groups, *p_(home, 1 min)_ = .013, *p_(home, 15 min)_ = .023, *p_(home, 24 hr)_ = .041, *n* = 4~6/group]. RM two‐way ANOVA shows significant effects of KD (*F* = 21, *p* < .0010) and time (*F* = 7.5, *p* = .0004), and Sidak's test shows significant effects at home (*p* = .034) and 1 hr (*p* = .0006). (d) Time course of total CaMKIIα protein after stCFC learning in both AAV_Ct_ and AAV_KD_ mice [all *p* > .05, *n* = 4~6/group]. RM two‐way ANOVA shows no significant effects of KD, time, and their interaction, and Sidak's test shows no significance at all time points. (e) Time course of changes of p‐T286 CaMKIIα (50 kDa) after stCFC learning in both AAV_Ct_ and AAV_KD_ mice (p‐T286 50kDa/total CaMKIIα; *p_(home; Ct, vKD)_ = .019) [*p* values in AAV_Ct_ groups, **p_(home, 1 min)_ = .0066, **p_(home, 15 min)_ = .0014, *p_(home, 1 hr)_ = .037, *p_(home, 24 hr)_ = .011, *p_(1 min, 24 hr)_ = .027, *p_(15 min, 24 hr)_ = .014; *p* values in AAV_KD_ groups, all *p* > .05, *n* = 4~6/group]. RM two‐way ANOVA shows significant effects of time (*F* = 5.5, *p* = .0017) and interaction (*F* = 3.1, *p* = .027), and Sidak's test shows a significant effect at home (*p* = .0014). (f) Time course of changes of p‐T305 CaMKIIα after stCFC learning in both AAV_Ct_ and AAV_KD_ mice (p‐T305/total CaMKIIα; AAV_Ct_, *p_(home; Ct, vKD)_ = .050, *p_(1 hr; Ct, vKD)_ = .041) [*p* values in AAV_Ct_ groups, all *p* > .05; *p* values in AAV_KD_ groups, *p_(home, 1 min)_ = .011, *p_(1 min, 15 min)_ = .016, *p_(1 min, 1 hr)_ = .043, *p_(1 min, 24 hr)_ = .024, *n* = 4~6/group)]. RM two‐way ANOVA shows no significant effect of KD, time, and their interaction, and Sidak's test shows no significance at all time points

We have shown that stCFC causes a reduction of vHC densin of control mice one day later (Figure 2b). To find how postshock densin was affected for 24‐hr period, the amount of densin was examined at the following time intervals: 1 min, 15 min, 1 hr, and 24 hr postshock (Figure 6a,b). Western gel band intensity was normalized to that of GAPDH, and the average intensity of home cage control group was set as 100%. Results showed a biphasic change of densin levels, where an immediate postshock drop by ~40% was followed by a recovery at 1 hr (~97%) and then by another reduced phase at 24 hr (~68%) (Figure 6c), which was consistent with the result in Figure 2b. vHC densin KD reduced the basal densin to ~68% of control mouse, and stCFC further dropped densin immediately by ~54% (i.e., ~31% of control basal), which was then recovered slowly to ~76% of KD basal level (i.e., ~52% of control basal) at 24 hr. We find that stCFC induces dynamic temporal changes of densin level for 24‐hr postshock period and the recovery of densin is insignificant in KD mice as expected.

### 3.9 Densin KD downregulates both p‐T286 and p‐T305 CaMKIIα, and stCFC also decreases p‐T286 CaMKIIα but increases p‐T305 CaMKIIα

To gain a clue on the molecular signaling pathway of densin in stCFC learning and memory processes, the phosphorylation status of CaMKIIα was examined in vHC densin KD (Figure 6). Phosphorylated CaMKIIα in vHC samples was analyzed at the same time points as densin was. First of all, the total CaMKIIα level was not affected either by densin KD procedure, shown at home cage data in Figure 6d, consistent with KO mice results (Carlisle et al., 2011), or by stCFC up to the following 24‐hr period (Figure 6d). The results suggest that the total CaMKIIα level is modulated independently from either densin or stCFC stimulus. Then, we measured p‐T286 or p‐T305 CaMKIIα in basal state and after stCFC. In control mice, stCFC caused dynamic changes of p‐T286 CaMKIIα level (Figure 6e). p‐T286 CaMKIIα was reduced to ~18% of basal level within a minute and recovered partially at 1 hr (~80%), which was then maintained up to 24 hr (~77%). Postshock correlative regulations on both p‐T286 CaMKIIα and densin seem to deviate later, because densin was decayed again at 24 hr (Figure 6c,e). In KD mice, the basal p‐T286 CaMKIIα was reduced to about half of control (Figure 6e), which was consistent with the result from KO mice (Carlisle et al., 2011). These results suggest a correlative change between densin and p‐T286 CaMKIIα in basal state. When stCFC was given, the early sharp decay of p‐T286 CaMKIIα in control was mostly gone in KD mice and its subsequent levels were not much different from controls up to 24 hr. stCFC‐induced peak reduction of p‐T286 CaMKIIα was insignificant in KD mice, but the reduction was still about half of control (~40% in KD vs. ~82% in control), a similar ratio to the ratio of basal densin levels (~50% in KD vs. 100% in control). Therefore, the basal densin level seems to be correlated with the amount of basal p‐T286 CaMKIIα in addition to the portion of p‐T286 CaMKIIα which is dephosphorylated after stCFC.

In general, inhibitory T305 phosphorylation follows T286 autophosphorylation of CaMKIIα (Hanson & Schulman, 1992; Patton et al., 1990). Since the basal p‐T305 CaMKIIα was also decreased in KD mice (by ~44%), the amount of basal densin also seems to correlate with the amount of p‐T305 CaMKIIα. stCFC learning increased p‐T305 CaMKIIα 1 hr later (~162%), which was maintained above the basal level up to 24 hr (Figure 6f). In KD mice, on the contrary, stCFC immediately increased p‐T305 CaMKIIα (~131%), which was then quickly decayed back to the basal level. Consequently, densin KD reduces the effects of stCFC on both densin and p‐T286 CaMKIIα and causes a faster transient increase of p‐T305 CaMKIIα.

## 4 DISCUSSION

We have studied a role of densin in CFC learning and memory processes. The results show that both basal p‐T286 and p‐T305 CaMKIIα are positively correlated with the amount of basal densin and suggest that proper regulations of both CaMKIIα activity and the amount of vHC densin seem important for the stCFC memory formation.

### 4.1 It takes an hour or so for stCFC memory to be formed

Analysis of freezing behaviors of control mice revealed that, after a CFC shock was given, it took at least an hour for freezing behavior to reach a peak response, which went down at 2 hr postshock (Figure 4g) and up one day later (Figure 3b). Even though vHC densin KD mice could show a similar freezing response as control mice at 1 hr postshock, their freezing was disappeared from 2‐hr to 24‐hr postshock periods. The results suggest that 1 ~ 2 hr of postshock period is the crucial time window for the formation of stable fear memory in control mice and further suggest a critical role of vHC densin during that time window.

### 4.2 stCFC induces dynamic temporal regulations of VHC densin

When a memory is formed, it is transformed through specific processes which are necessary for the consolidation of the memory and its reactivation destabilizes the pre‐existing postsynaptic structures of memory engram neural circuits, which results in a disruption or reconsolidation of the memory, or an update with a new memory (Alberini et al., 2006; Dudai & Eisenberg, 2004; Finnie & Nader, 2012; Nadel et al., 2012; Tronson & Taylor, 2007). It is thought that the postsynaptic side of a synapse at its basal state exists in a homeostatically stable molecular architecture, which densin is a part of, and a new stCFC learning needs to disrupt this pre‐existing structure before making a new architecture for the new memory formation. Moreover, protein degradation is considered upstream of the protein synthesis for long‐term memory storage and stability (Jarome & Helmstetter, 2013; Jarome & Helmstetter, 2014; Lip et al., 2017).

Interactions among densin, CaMKIIα, Ca_v_1.3 channels, and PDZ proteins can regulate postsynaptic organization and stability (Kennedy, 1998; Ko & Kim, 2007; Penny & Gold, 2018; Quitsch et al., 2005; Sanhueza & Lisman, 2013; Stanika et al., 2015; Vessey & Karra, 2007). We find that densin is highly expressed in vHC compared with dHC (Figure 1) and only the vHC densin is reduced one day after stCFC in control mice (Figure 2). On closer observation, after stCFC, vHC densin was reduced immediately, recovered close to the basal level 1 hr later, and decayed back again 24 hr later (Figure 6c). Therefore, the immediate reduction of densin may be due to the degradation of densin, which will then facilitate the disruption of the existing postsynaptic structure, making it available for the next memory formation (Almeida‐Correa & Amaral, 2014; Jarome et al., 2016; Jarome et al., 2013; Lynch et al., 2015). By the same token, densin in the stable basal architecture would work inherently against any structural changes necessary for the new learning. Therefore, some reduction of densin by KD may help make pre‐existing architectures unstable or easily disrupted and more readily available for building new structures for storing the new stCFC memory. This idea could explain the very early transient enhancement of freezing response in KD mice (Figure 4b). However, freezing was normal up to ~1 hr postshock except that very early period in KD mice (Figure 4g), suggesting that densin is not critical, or the residual densin is enough, for expressing freezing behaviors until ~1 hr postshock. Then, KD mice, which had about half of control densin up to ~1 hr postshock but the same level of control at 24 hr, failed to show freezing behaviors at 2 hr postshock, implicating that densin ≤1 hr postshock, which is as large as the control basal level, is important for the later stable stCFC memory formation, not directly for freezing at 1 hr postshock (Figures 4g and 6c). Based on these results, we postulate that a certain amount of densin ≤1 hr postshock is necessary for rebuilding the postsynaptic molecular structure or stabilizing the stCFC memory after learning‐induced signaling pathways disrupt the pre‐existing postsynaptic architecture.

In terms of the functional meaning of 1 ~ 2‐hr time window, it may indicate the time required for making new proteins for memory formation. When memories are formed and stabilized, they require protein synthesis and gene regulation (Chew et al., 1996; Freeman et al., 1995; Grecksch & Matthies, 1980; Jarome & Helmstetter, 2014; Katche et al., 2013; Schafe et al., 1999). For example, Bourtchouladze et al. (1998) showed that the consolidation of CFC memory required PKA‐dependent and protein translation‐dependent processes which occurred 1 ~ 3 hr of postshock period. Fulton et al. (2005) also found that protein translation for the appetitive conditioning long‐term memory occurred ~1 hr after the trial learning. Furthermore, Rudy and Matus‐Amat (2005) showed that long‐term memory formation required the translation activity of vHC area 1 hr after stCFC learning. Therefore, it is plausible that 1 ~ 2 hr of postshock time window is the time required for vHC densin translation or new transcriptional activity for stCFC memory formation and/or its retrieval, which appears as the recovery phase of densin. Additionally, since densin is recovered fully at ~1 hr postshock in control mice, it further implicates that the basal densin level is already homeostatically regulated to keep it optimal for participating in stCFC learning and memory formation process. Thus, to check this idea, it will be interesting to see how the basal densin level is regulated especially when vHC neurons need to store consecutive memories from diverse learning stimuli.

Here, we have not observed immediate postshock freezing in control mice (Figure 4b). Since rat showed immediate postshock freezing, in which extrahippocampal areas such as ventral periaqueductal gray and amygdala were involved (Kim et al., 1993), the possibility of difference in species and/or neural circuits for the emergence of postshock freezing remains to be studied.

### 4.3 stCFC induces dynamic temporal regulations of both p‐T286 and p‐T305 CaMKIIα

We found that stCFC caused immediate reductions of both densin and p‐T286 CaMKIIα and a 1‐hr‐late increase of p‐T305 CaMKIIα in vHC of control mice. The immediate drop of p‐T286 CaMKIIα and densin might facilitate their dissociation from the postsynaptic molecular structure and its successive breakup. Increase of inhibitory p‐T305 CaMKIIα 1 hr postshock was coincident with the time of densin recovery as well as the stCFC memory formation. This enhancement of p‐T305 CaMKIIα was gone in vHC densin KD mice. Therefore, the increment of p‐T305 CaMKIIα 1 hr postshock may be also important for the stCFC memory formation. Since these changes of phosphorylation status at both sites of CaMKIIα are expected to reduce CaMKIIα activity, the early process of stCFC learning may need a suppression of CaMKIIα activity in control mice. Moreover, the 1‐hr postshock recovery of p‐T286 CaMKIIα was still below the basal level and was maintained up to 24 hr. Therefore, CaMKIIα activity after stCFC learning seems negatively correlated to a degree with stCFC memory storage and/or retrieval one day later (*p_(home, 24 hr)_ = .011).

In regard to the role of p‐T286 CaMKIIα in behavioral cognitive effects, p‐T286 CaMKIIα is related to hippocampal‐dependent learning but not necessarily to memory storage (Giese et al., 1998; Giese & Mizuno, 2013; Irvine et al., 2006). Lepicard et al. (2006) showed that CFC caused up‐regulation of CaMKIINα mRNA, an endogenous CaMKIIα inhibitor, 30 ~ 60 min after stCFC, which would reduce CaMKIIα activity. Moreover, Buard et al. (2010) showed that p‐T286 CaMKIIα is not important for storage or retrieval of mouse stCFC memory 24 hr later using a selective peptide inhibitor, tatCN21. Additionally, Naskar et al. (2014) showed that p‐T286 CaMKIIα was reduced 6 hr after an appetitive conditioning learning in snail and an increase of p‐T305 CaMKIIα was important for long‐term memory formation of the associative classical conditioning.

One idea worth mentioning is that although many studies have shown that the activation of CaMKIIα or the expression of p‐T286 CaMKIIα is necessary for the memory formation as well as synaptic plasticity processes, it has been unclear whether the activation of CaMKIIα itself is crucial or the optimal amount of p‐T286 CaMKIIα is critical for those processes. In this study, one plausible cause of the impairment of stCFC memory formation in vHC densin KD mice may simply be due to the significant reduction of the basal p‐T286 CaMKIIα level itself, which is below a threshold level required for participating in stCFC memory formation processes. Miller et al. (2002) showed that 24‐hr CFC memory retention and late LTP were impaired in a mutant mice, where basal PSD CaMKIIα was reduced to ~20% of control. This result may accord with the idea of the basal CaMKIIα threshold level for stCFC memory formation, too. In this study, densin also seems to fit with the threshold idea, where a critical amount of densin is necessary for the consolidation of stCFC learning and memory. Besides, since dtCFC memory formation occurs in vHC densin KD mice, it will be interesting to know how this threshold idea is transformed while forming dtCFC memory.

### 4.4 Known interactions among densin, CaMKIIα and others, and their functional implications during stCFC

Densin can bind either to unphosphorylated form of CaMKIIα and inhibit CaMKIIα activation, or to p‐T286 form of CaMKIIα, which has a higher affinity for densin (Jiao et al., 2011; Strack et al., 2000; Walikonis et al., 2001). On the other hand, S1397 phosphorylation of densin by CaMKIIα reduces the affinity for CaMKIIα (Walikonis et al., 2001). Moreover, autophosphorylation of T305 site of CaMKIIα reduces the catalytic activity via interrupting Ca^2+^/CaM binding and facilitating dephosphorylation of p‐T286 (Colbran & Soderling, 1990; Hashimoto et al., 1987; Lou & Schulman, 1989; Patton et al., 1990). Thereby, it is likely that reduced binding of p‐T305 CaMKIIα with densin promotes its dissociation from PSD and results in impairments of LTP and learning (Elgersma et al., 2002; Shen et al., 2000; Strack et al., 1997).

In addition, densin may affect CaMKIIα functions via regulations of phosphatase activities, which are important for the timely resetting of CaMKIIα activity for synaptic plasticity and memory processes (He et al., 2016; Lisman & Zhabotinsky, 2001; Pagani & Merlo, 2019). Type I protein phosphatase (PP1) can interact with densin via spinophilin (Baucum et al., 2010) and dephosphorylates p‐T286 site of CaMKIIα, KD of hippocampal PP1 enhances CFC memory formation (Shields et al., 1985; Strack et al., 1997; Peters et al., 2009; but see Yang et al., 2015), and CFC induces the transcriptional silencing of type I protein phosphatase (PP1) gene (Miller & Sweatt, 2007). Moreover, another phosphatase, phosphatase and tensin homolog α (PTENα) can bind to the N‐terminal side of CaMKIIα and resets its activity by dephosphorylating p‐T305/306 CaMKIIα sites, and loss of PTENα impairs stCFC memory formation (Wang, Mei, et al., 2017). All these results give an idea of dynamic interactions between densin and CaMKIIα such that densin can bind to CaMKIIα at basal state and stimuli inducing T286 autophosphorylation of CaMKIIα will enhance their binding, and either phosphorylation of densin by CaMKIIα or T305 autophosphorylation of CaMKIIα will weaken the interaction. Since the total CaMKIIα is not changed after stCFC in this study, the ratio of phosphorylated and unphosphorylated forms of CaMKIIα and their binding affinities to densin will change inversely and dynamically during CFC learning and memory process.

Regarding the role of densin interaction with CaMKIIα, densin KO mice study seems to exclude a PSD localization role of densin after stimulus or learning (Carlisle et al., 2011; Shen et al., 2000; Strack et al., 1997). Since densin makes a ternary complex of CaMKIIα and Ca_v_1 channels, densin can help CaMKIIα get close to Ca_v_1‐type channels, which will facilitate T286 autophosphorylation of CaMKIIα, regulate channel trafficking to synapse and Ca^2+^ current, and produce a better excitation‐transcription coupling process that is necessary for synthesizing proteins for new memory formation (Hudmon et al., 2005; Jenkins et al., 2010; Ma et al., 2015; Stanika et al., 2016; Walikonis et al., 2001; Wang, Marks, et al., 2017; Wang, Mei, et al., 2017; Wheeler et al., 2008, 2012). In structural terms, densin is also known to increase CaMKIIα binding to α‐actinin, forming another ternary structure, and T286A or T305D CaMKIIα mutant, which has reduced binding to α‐actinin as well as NMDAR, exists less in PSD and shows little freezing responses of stCFC 24 hr later and impaired LTP (Elgersma et al., 2002; Leonard et al., 2002; Park et al., 2008; Robison et al., 2005; Shen et al., 2000). Interactions of densin with shank and δ‐catenin and their functions in dendritic growth have been shown (Quitsch et al., 2005). Hence, densin KD together with reduction of both p‐T286 and p‐T305 CaMKIIα may disrupt the postsynaptic structural organization and disturb the downstream paths of Ca^2+^ signaling, leading to the impairments of synaptic plasticity and stCFC memory stabilization and retrieval.

### 4.5 Differential roles of ventral versus dorsal hippocampal densin in single‐ and double‐trial CFC memory formation and retrieval

We showed that densin was expressed differentially along the septo‐temporal hippocampal axis and only vHC densin KD impaired Day 1 stCFC memory retention. We further found that vHC densin KD partially blocked dtCFC memory formation, suggesting a different mechanism of dtCFC compared to that of stCFC. Interestingly, repeated CFC trials and repeated trial learnings disinhibited the inhibitory effect of T286A CaMKIIα on CFC memory formation (Irvine et al., 2005) and the inhibitory effect of kinase‐dead K42R CaMKIIα mutant on the inhibitory avoidance memory formation (Yamagata et al., 2009), respectively.

In particular, here dtCFC revealed incremental patterns of freezing responses during 24‐hr memory retention test in two respective comparison cases. One case is between stCFC and dtCFC within vHC control mice (Figure 3b,d, open circles). Here, only dtCFC memory test shows the incremental retrieval of freezing memory. Besides, a similar incremental retrieval kinetics of dtCFC memory appeared in vHC densin KD mice, although the average freezing level was half of control (Figure 3d), suggesting that dtCFC learning initiates a pathway where vHC region is involved in such an incremental retrieval process of dtCFC memory. Since dtCFC memory recovers during the test in KD mice, dtCFC may be strong enough to utilize the residual densin proteins from incomplete KD efficiency for the dtCFC memory formation and retrieval process. Consistent with this idea, we observed that stCFC with a stronger shock intensity such as 0.7 mA, rather than 0.5 mA, could form a stCFC memory in densin KD mice (Figure S3B,C), though more sensory effects need to be tested. It will be interesting to know whether dtCFC memory in KD mice can be restored as the same as in control mice, if a longer test period were given. The result will tell much more clearly the role of vHC densin in dtCFC memory formation and retrieval.

The other case is the interregional comparison of dtCFC memory retrieval between vHC and dHC (Figure 3d,e, open circles). Considering damages done commonly to vHC or dHC region by the control KD process, the result further suggests that vHC region has a role in the incremental retrieval of dtCFC memory compared to dHC region. In dHC densin KD mice, freezing behaviors of both stCFC and dtCFC memory tests showed a‐minute‐long quick recoveries, implying a role of dHC densin in the very early retrieval period of both CFC memories (Figure 3c,e). In brief, the results of dtCFC reveal a role of vHC region in generating the incremental retrieval of dtCFC memory and also uncover a role of dHC densin in expressing the very early freezing behaviors during retrieval of both CFC memories.

## 5 CONCLUSION

In this study, a role of postsynaptic densin, interacting with many postsynaptic molecules including CaMKIIα, was studied during CFC learning and memory processes using shRNA viral knockdown approach. Though an incomplete KD efficiency as well as inhomogeneous neuronal cell types targeted could limit our discussion on the role of densin in CFC learning and memory process, KD of subregional hippocampal densin affected the contextual fear learning and memory formation and retrieval processes differentially depending on the type of stimulus pattern or its location within hippocampus.

## CONFLICT OF INTEREST

The authors have declared that there exists no conflict of interest to declare.

## AUTHOR CONTRIBUTIONS

CHK conceived the work, drafted and wrote the article, and is responsible for the intellectual content. SK contributed to the design of the work and acquired and analyzed the data. SHK and HJ contributed to the animal behavior experiments, and JR and BS contributed to western blot experiment and data analysis.

### Peer Review

The peer review history for this article is available at https://publons.com/publon/10.1002/brb3.1891.

## Supporting information

Figure S1

Figure S2

Figure S3

Supplementary Material

## Data Availability

We declare that the data that support the findings of this study are available from the corresponding author upon reasonable request.
