# Supplementary figures and images for: Role of densin‐180 in mouse ventral hippocampal neurons in 24‐hr retention of contextual fear conditioning
Source: Brain Behav. 2020 Oct 16;10(12):e01891. doi: 10.1002/brb3.1891 (PMC7749528; doi:10.1002/brb3.1891)

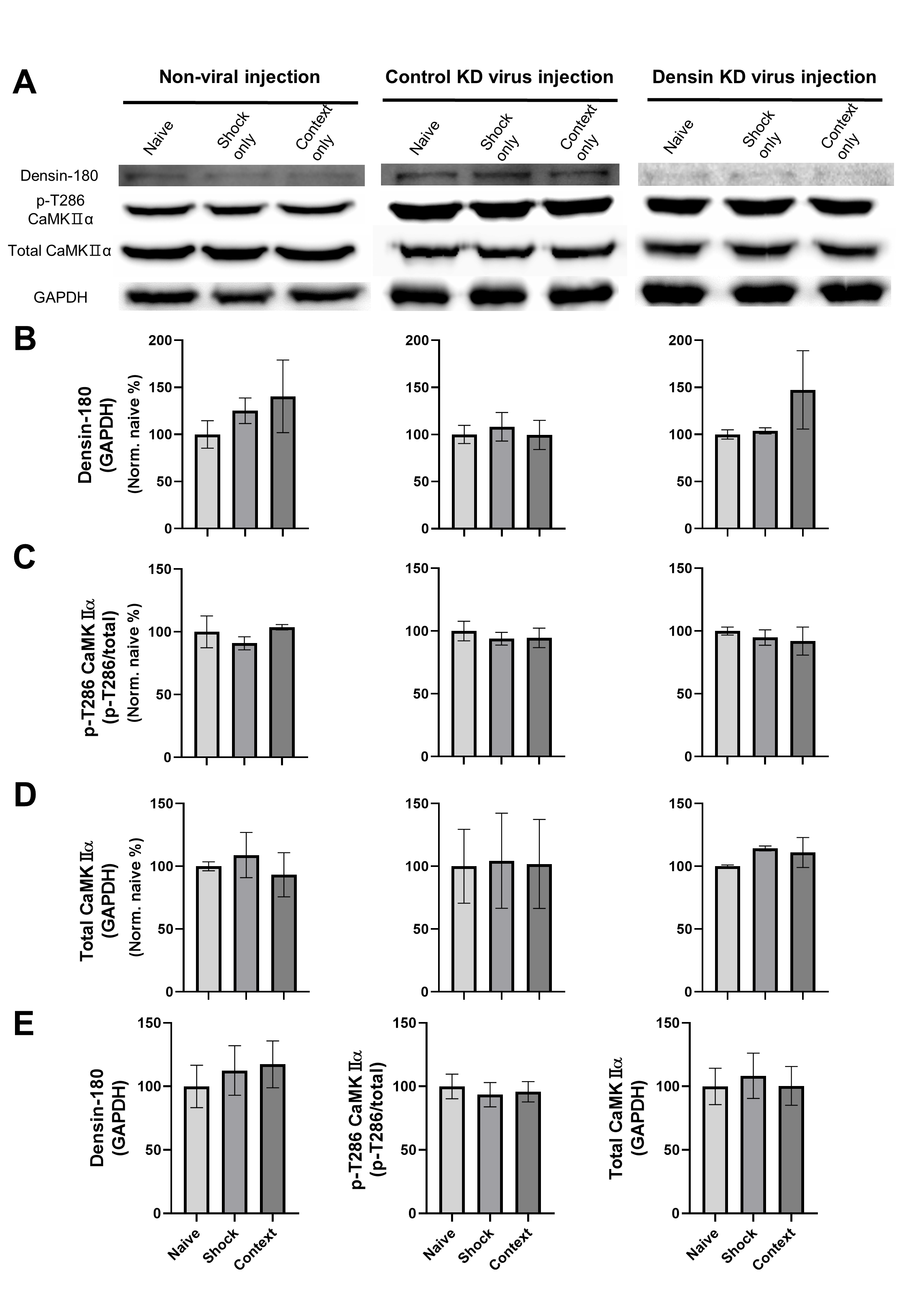

Supplement: Supplementary file 1 — Figure S1 [file BRB3-10-e01891-s001.tif]

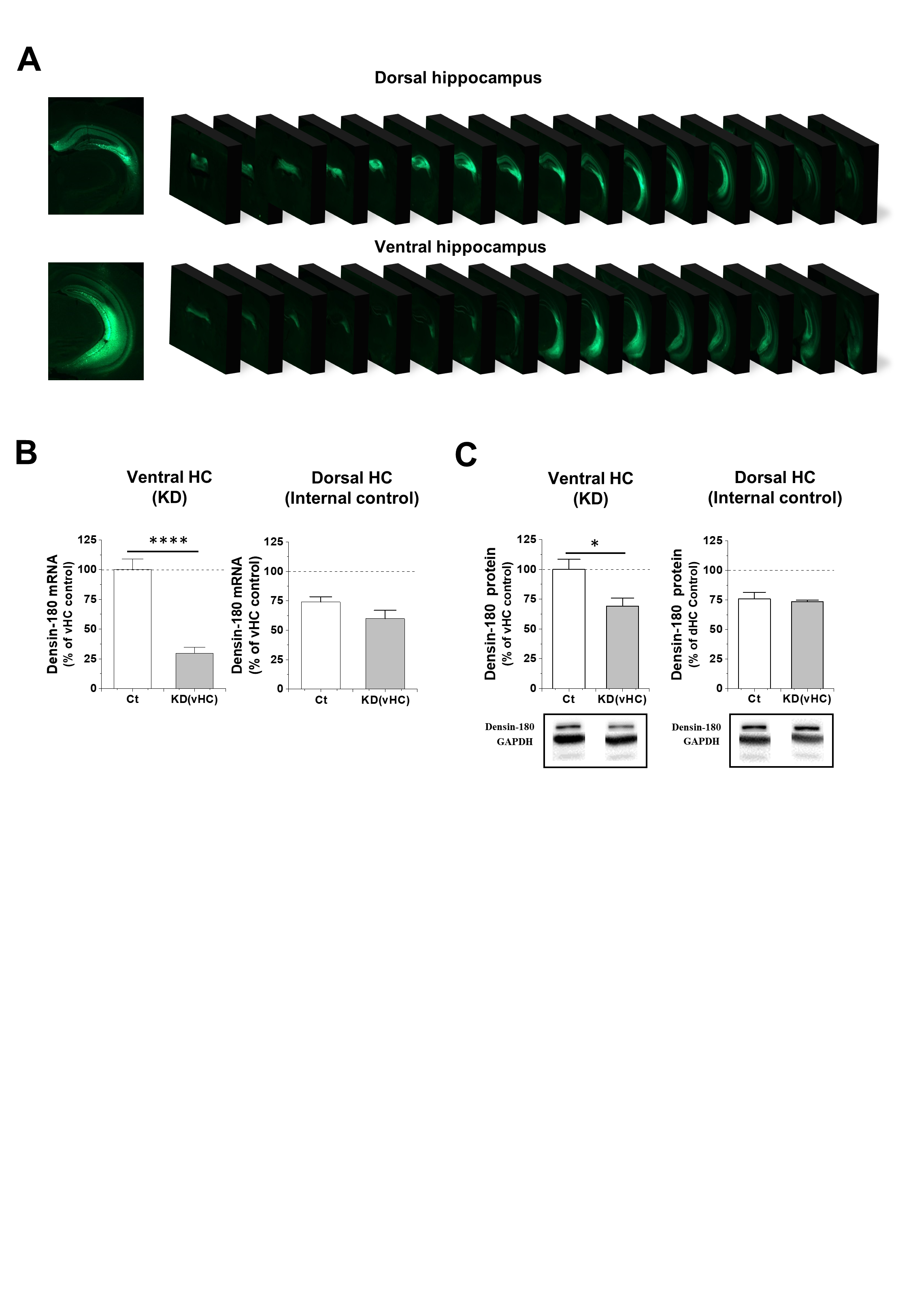

Supplement: Supplementary file 2 — Figure S2 [file BRB3-10-e01891-s002.tif]

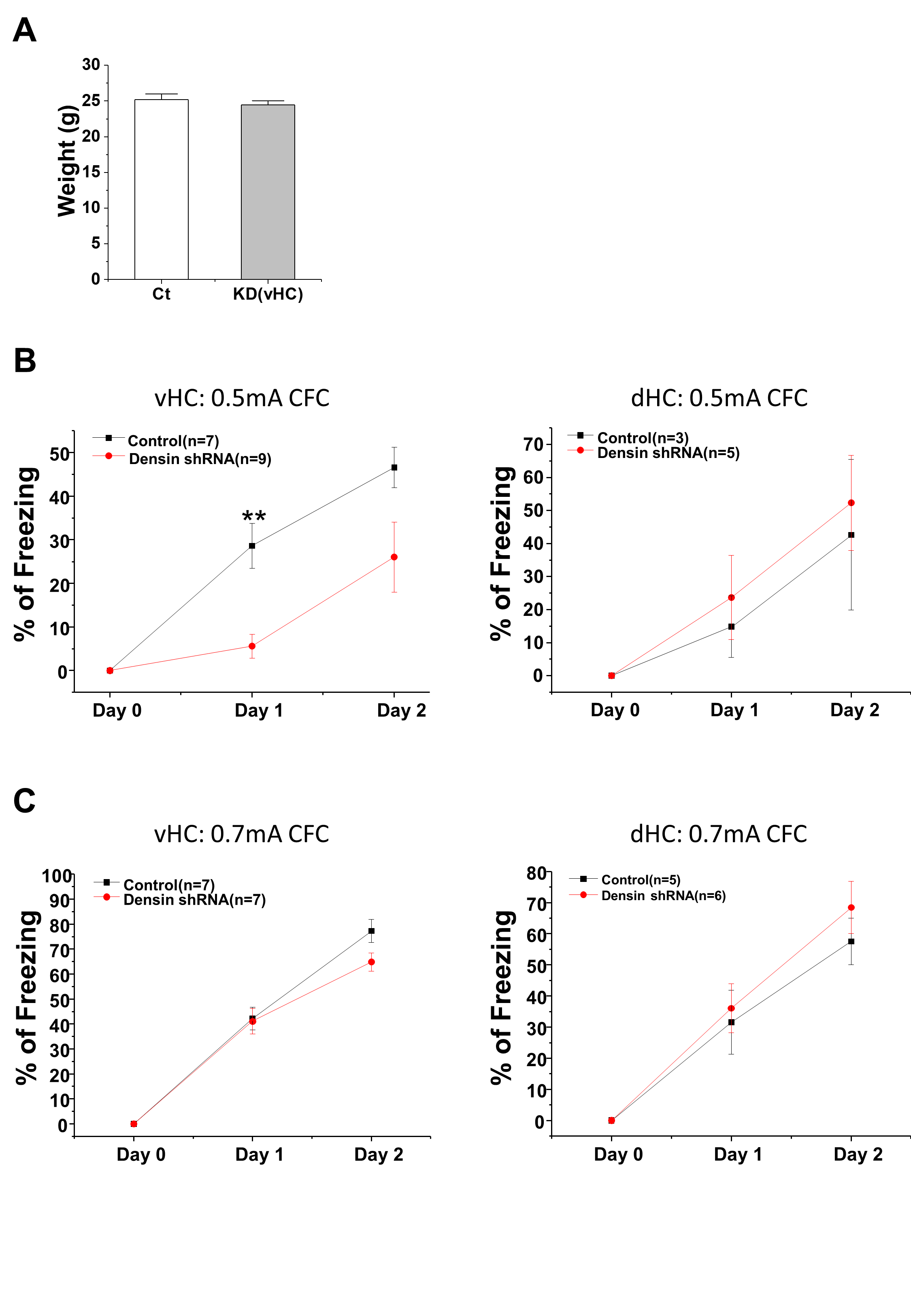

Supplement: Supplementary file 3 — Figure S3 [file BRB3-10-e01891-s003.tif]
