# Supplementary material for: Role of densin‐180 in mouse ventral hippocampal neurons in 24‐hr retention of contextual fear conditioning
Source: Brain Behav. 2020 Oct 16;10(12):e01891. doi: 10.1002/brb3.1891 (PMC7749528; doi:10.1002/brb3.1891)
Supplement: Supplementary file 4 — Supplementary Material [file BRB3-10-e01891-s004.docx]

**Supplementary Figure 1. Comparisons of densin, CaMKⅡα and p-T286 CaMKⅡα expressions among naïve and non-associated controls (shock or context only) in each of non-viral infected, control KD virus infected, and densin KD virus infected mice.**

We performed experiments using three independent mice groups (non-viral injected group, control KD virus injected group, densin KD virus injected group). (A) Expressions of densin, total CaMKⅡα, and phospho-T286 CaMKⅡα were measured in three differently conditioned mice such as naïve home cage, shock-only (0.5 mA/2 sec), context-only (3 min exploration). 20 μg protein sample/each lane. Protein bands of densin and CaMKⅡα were normalized by GAPDH protein intensity and phospho-T286 CaMKⅡα band was normalized by total CaMKⅡα, which were converted into percentage (%). (B) Densin-180 expression levels in each mouse groups (non-infected group, naïve, 100.00 ± 14.51%, shock only, 125.23 ± 13.58%, context only, 140.47 ± 38.58%, n = 2; control KD virus infected group, naïve, 100.00 ± 9.65%, shock only, 108.18 ± 15.18%, context only, 99.50 ± 15.55%, n = 3; densin KD virus infected group, naïve, 100.00 ± 14.51%, shock only, 125.23 ± 13.58%, context only, 140.47 ± 38.58%, n = 2. (C) Phospho-T286 CaMKⅡα expression levels in each mice groups (non-infected group, naïve, 100.00 ± 12.73%, shock only, 90.91 ± 5.18%, context only, 103.73 ± 2.12%, n = 2; control KD virus infected group, naïve, 100.00 ± 7.75%, shock only, 93.87 ± 5.00%, context only, 94.56 ± 7.71%, n = 3; densin KD virus infected group, naïve, 100.00 ± 3.13%, shock only, 94.84 ± 6.13%, context only, 92.05 ± 11.18%, n = 2). (D) Total CaMKⅡα expression levels in each mice groups (non-infected group, naïve, 100.00 ± 3.58%, shock only, 108.93 ± 17.94%, context only, 93.26 ± 17.60%, n = 2; control virus infected group, naïve, 100.00 ± 29.49%, shock only, 104.38 ± 37.86%, context only, 101.81 ± 35.44%, n =3; densin KD virus infected group, naïve, 100.00 ± 1.01%, shock only, 114.27 ± 1.96%, context only, 110.98 ± 11.91%, n = 2). (E) Sum data from all the results of three independent mice groups. (densin, naïve, 100.00 ± 16.72%, shock only, 112.58 ± 19.53%, context only, 117.39 ± 18.44%, n = 7; p-T286 CaMKⅡα, naïve, 100.00 ± 9.74%, shock only, 93.55 ± 9.57%, context only, 95.77 ± 7.99%, n = 7; total CaMKⅡα, naïve, 100.00 ± 14.32%, shock only, 108.37 ± 17.79%, context only, 100.44 ± 15.25%, n = 7). Using two-tailed t-test, all *p* values generated above B)-E) conditions were non-significant (>>0.05).

**Supplementary Figure 2. Verification of AAV densin shRNA expression and efficiency of densin knockdown in dHC and vHC of mouse.**

(A) The expression pattern of GFP in the dHC and vHC 2 week after AAV injection (Upper; vHC, bottom; dHC). The coronal hippocampal sections were made. See Methods. Two-tailed t-test at α = 0.05 unless otherwise mentioned.

(B) Knockdown of densin mRNA expression in vHC injection mice. KD efficiency was normalized over that of GFP AAV injected into the vHC. Left panel, vHC injection mice (AAV_Ct_, 100.0 ± 9.1%, n = 12; AAV_KD,_ 29.8 ± 4.9%, n = 12, **** p < 0.0001). Right panel, dHC internal control (AAV_Ct_, 74.0 ± 4.5%, n = 12; AAV_KD,_ 59.9 ± 7.1%, n = 12, p > 0.05). GAPDH mRNA was used as an internal control of mRNA expression.

(C) Knockdown of densin protein level in vHC injection mice. KD efficiency was normalized over that of GFP AAV injected into the vHC. Left panel, vHC injection mice. (AAV_Ct_, 100 ± 8.5%, n = 4; AAV_KD_, 69.3 ± 6.7%, n = 4, * p < 0.05). Right panel, dHC internal control. (AAV_Ct_, 75.9 ± 5.6%, AAV_KD_, 73.4 ± 1.5%, n = 4, p > 0.05). GAPDH protein was used as an internal control of protein expression.

**Supplementary Figure 3. Examples of mouse weight measurements and comparison of 0.5 mA vs 0.7 mA unconditional shock stimulus effects on stCFC and dtCFC.**

(A) An example of weight measurements of vHC control virus and KD virus injected mice. Ct, 25.16 ± 0.84 g, n = 7; KD, 24.44 ± 0.58 g, n = 8, *p* = 0.24

(B) Freezing (%) measurements of basal level (Day 1), 24 hr after stCFC (Day 2), and 24 hr after dtCFC (Day3) with 0.5 mA shock intensity as unconditioned stimulus in dHC and vHC control and KD mice groups. Left panel, vHC injection mice (AAV_Ct_, Day 1 (basal), 0.0 ± 0.0%, n = 7; AAV_KD,_ Day 1 (basal), 0.0 ± 0.0%, n = 9; Day 1, p > 0.05, Day 2, p = 0.001104; Day 3, p = 0.078366). Right panel, dHC injection mice (AAV_Ct_, Day 1 (basal), 0.0 ± 0.0%, n = 3; AAV_KD,_ Day 1 (basal), 0.0 ± 0.0%, n = 5; Day 1, Day 2, Day 3, p > 0.05).

(C) Freezing (%) measurements of basal level (Day 1), 24 hr after stCFC (Day 2), and 24 hr after dtCFC (Day3) with 0.7 mA shock intensity as unconditioned stimulus in dHC and vHC control and KD mice groups. Left panel, vHC injection mice (AAV_Ct_, Day 1 (basal), 0.0 ± 0.0%, n = 7; AAV_KD,_ Day 1 (basal), 0.0 ± 0.0%, n = 7; Day 1, Day 2, Day 3, p > 0.05). Right panel, dHC injection mice (AAV_Ct_, Day 1 (basal), 0.0 ± 0.0%, n = 5; AAV_KD,_ Day 1 (basal), 0.0 ± 0.0%, n = 6; Day 1, Day 2, Day 3, p > 0.05).
